# Family Caregiver Needs and Preferences for Virtual Training to Manage Behavioral and Psychological Symptoms of Dementia: Interview Study

**DOI:** 10.2196/24965

**Published:** 2021-02-10

**Authors:** Magaly Ramirez, Miriana C Duran, Chester J Pabiniak, Kelly E Hansen, Ann Kelley, James D Ralston, Susan M McCurry, Linda Teri, Robert B Penfold

**Affiliations:** 1 Department of Health Services University of Washington School of Public Health Seattle, WA United States; 2 Kaiser Permanente Washington Health Research Institute Seattle, WA United States; 3 Child, Family, and Population Health Nursing University of Washington School of Nursing Seattle, WA United States; 4 University of Washington School of Nursing Seattle, WA United States

**Keywords:** dementia, Alzheimer disease, behavioral symptoms, caregivers, internet-based intervention, education, behavior, symptom, psychology, qualitative, caregiver, intervention, training, virtual care, digital health

## Abstract

**Background:**

Behavioral and psychological symptoms of dementia (BPSD) are associated with increased stress, burden, and depression among family caregivers of people with dementia. STAR-Caregivers Virtual Training and Follow-up (STAR-VTF) is adapted from an evidence-based, in-person program that trains family caregivers to manage BPSD. We used a human-centered design approach to obtain feedback from family caregivers about STAR-VTF. The program will be evaluated using a pragmatic randomized trial.

**Objective:**

The objective of the study was to understand the needs of family caregivers for improving BPSD management and the extent to which caregivers perceived that STAR-VTF could address those needs.

**Methods:**

Between July and September 2019, we conducted 15 semistructured interviews with family caregivers of people with dementia who receive care at Kaiser Permanente Washington in the Seattle metropolitan area. We identified participants from electronic health records, primarily based on a prescription for antipsychotic medication for the person with dementia (a proxy for caregivers dealing with BPSD). We showed caregivers low-fidelity prototypes of STAR-VTF online self-directed materials and verbally described potential design elements. We obtained caregiver feedback on these elements, focusing on their needs and preferences and perceived barriers to using STAR-VTF. We used a hybrid approach of inductive and deductive coding and aggregated codes to develop themes.

**Results:**

The idea of a virtual training program for learning to manage BPSD appealed to caregivers. They said health care providers did not provide adequate education in the early disease stages about the personality and behavior symptoms that can affect people with dementia. Caregivers found it unexpected and frustrating when the person with dementia began experiencing BPSD, symptoms they felt unprepared to manage. Accordingly, caregivers expressed a strong desire for the health care organization to offer programs such as STAR-VTF much sooner. Caregivers had already put considerable effort into problem solving challenging behaviors. They anticipated deriving less value from STAR-VTF at that point. Nonetheless, many were interested in the virtual aspect of the training due to the convenience of receiving help from home and the perception that help from a virtual program would be timelier than traditional service modalities (eg, face to face). Given caregivers’ limited time, they suggested dividing the STAR-VTF content into chunks to review as time permitted. Caregivers were interested in having a STAR-VTF provider for additional support in managing challenging behaviors. Caregivers reported a preference for having the same coach for the program duration.

**Conclusions:**

Caregivers we interviewed would likely accept a virtual training program such as STAR-VTF to obtain information about BPSD and receive help managing it. Family caregivers anticipated deriving more value if STAR-VTF was offered earlier in the disease course.

## Introduction

Alzheimer disease and related dementias (ADRD) are irreversible, progressive brain disorders that eventually affect a person’s ability to perform basic activities, including bathing, feeding, and dressing. ADRD is the fifth leading cause of death among people 65 years and older in the United States [1]. The US prevalence of ADRD is projected to nearly triple from 5 million in 2014 to 13.9 million in 2060 [2]. People with dementia require high levels of care, most of which is provided by informal caregivers such as spouses and adult children. Up to 90% of people with dementia will experience behavioral and psychological symptoms of dementia (BPSD) over the course of their illness [3]. BPSD that can be particularly challenging to family caregivers include agitation, anxiety, irritability, depression, delusions, hallucinations, and sleep changes. BPSD are associated with increased stress, burden, and depression among family caregivers of people with dementia [4,5].

STAR-Caregivers (STAR-C) is a nonpharmacological intervention endorsed by the US Department of Health and Human Services Administration on Aging in which a coach trains family caregivers over multiple face-to-face sessions on how to manage BPSD. STAR-C is demonstrated to reduce caregiver burden, caregiver depression, and the frequency and severity of BPSD [6]. The STAR-C training is 6 sessions, covering topics such as expectations about ADRD, communication with a person with ADRD, strategies for dealing with BPSD, and coping with caregiving, including pleasant activities for caregivers and patients. In its original form, coaches conduct in-person weekly sessions with caregivers in their homes with follow-up telephone calls. Despite evidence to support its efficacy, STAR-C has not been widely disseminated across health care and community settings, in part due to the cost of providing in-person training and using printed materials [7-9].

To address these implementation barriers, a Kaiser Permanente Washington Health Research Institute pragmatic trial is testing the feasibility of STAR-C Virtual Training and Follow-up (STAR-VTF) (ClinicalTrials.gov NCT04271046) [10], which will deliver the training to family caregivers virtually. Caregivers will complete online training modules asynchronously, have 30-minute weekly telephone check-ins with a coach (social worker or mental health counselor) at Kaiser Permanente Washington (KPWA), and have ongoing support from the coach via secure email messages within the KPWA member portal. Caregivers will access the online training modules via a website hosted on the Kaiser Permanente School of Allied Health Sciences extended learning management system. Caregivers will log in to the website from their preferred web browser using their email address and a user-generated password. While caregivers will not enter personal health information or other identifiable information, the website will automatically collect their IP addresses. The KPWA technology risk review team declared caregivers’ use of the website as minimal risk. Only caregivers living in Washington State that meet eligibility for the pragmatic trial will have access to the website.

While considerable evidence supports the efficacy of STAR-C, and implementation challenges from a health care system perspective could be addressed by changing the modality of program delivery from in person to virtual, family caregivers’ views on participating in a virtual training program are needed. A framework for using human-centered design to improve the implementation of evidence-based interventions recommends that early phases of the design process focus on gathering information to understand the viewpoints of all stakeholders [11]. This information is then used to iteratively design, build, and test solutions that directly address the needs and preferences of all stakeholders, particularly end users such as family caregivers in STAR-VTF. Human-centered design is widely considered the key to designing tools that end users will find useful and easy to use, factors that are associated with acceptance and actual use of tools [12].

Early in STAR-VTF development, when the original in-person training was being adapted for virtual delivery, our research team used a human-centered design approach to obtain feedback from family caregivers on the idea of a virtual training program. The objective was to understand the needs of family caregivers for improving BPSD management and the extent to which they perceived that a program such as STAR-VTF could address their needs. To achieve this, we conducted and analyzed 15 semistructured interviews with family caregivers of people with ADRD who receive care at KPWA in the Seattle metropolitan area. The COVID-19 pandemic has shed light on the urgent need to design and evaluate digital health strategies that offer support virtually [13], especially for families caring for vulnerable older adults. Effective digital health strategies to support family caregivers are critically needed during the pandemic and will remain important in the post–COVID-19 era.

## Methods

We conducted semistructured interviews with family caregivers of people with ADRD. The institutional review boards at Kaiser Permanente Washington Health Research Institute and the University of Washington approved the study. Study participants provided written informed consent.

### Recruitment

To identify potential study participants, we extracted data from the KPWA electronic health record (EHR) and administrative claims system to identify patients aged 65 years or older with an ADRD diagnosis and a new prescription for an antipsychotic medication within the past 2 years. A prescription for an antipsychotic medication was a proxy for identifying caregivers who may have struggled with managing BPSD. We excluded patients with an International Classification of Diseases Ninth Revision—Clinical Modification (ICD-9-CM) or ICD-10-CM diagnosis of bipolar disorder or schizophreniform disorder and those in assisted living or skilled nursing facilities. For patients meeting our criteria, we mailed a packet to their caregiver with a cover letter describing the goals of the study and a consent form. One week after the mailing, a study staff member phoned caregivers to invite study participation. The staff member attempted up to three calls with up to two voicemail messages. Caregivers interested in participating were screened for eligibility during the phone call. Caregivers were eligible if they were 21 years or older; were an adult child, spouse or partner, or close friend of the patient; lived with the patient (or within 5 miles); provided at least 8 hours of care per week; and lived in King, Snohomish, or Pierce counties, Washington. We excluded caregivers with a diagnosis of ADRD. For eligible, interested caregivers, the staff member scheduled a date, time, and location to conduct the interview.

### Data Collection

We conducted all interviews in person between July and September 2019. Interviews took place at a convenient location for caregivers, such as homes or KPWA facilities. Using a semistructured interview guide, we asked caregivers what challenging BPSD the patient experienced, how caregivers typically responded to BPSD, and how BPSD affected caregivers. Next, we used 2 storyboards to illustrate the potential experience of a caregiver using STAR-VTF to learn how to improve management of BPSD (Multimedia Appendix 1). The first storyboard depicted a caregiver struggling with behavioral symptoms in the person with dementia and learning about STAR-VTF through a health care provider. The second storyboard depicted a caregiver choosing which behavioral symptom to focus on, using STAR-VTF to learn strategies for responding to the symptom, and speaking on the phone with a coach for additional support. We asked caregivers questions to gauge their initial reactions to the idea of STAR-VTF and their interest in using the virtual program. Finally, we showed caregivers low-fidelity prototypes of the STAR-VTF online self-directed materials and verbally described potential design elements, including information content, visual and auditory presentation of information, and user interaction (Multimedia Appendix 2). We asked caregivers questions to obtain feedback on these design elements, elicit needs and preferences, and understand perceived barriers to using STAR-VTF.

We surveyed caregivers for sociodemographic and caregiving characteristics. Caregivers received US $100 for participating. All interviews were audiorecorded and transcribed verbatim by a professional transcription company. The transcripts were proofread by the interviewer. Interviews were 40 to 60 minutes.

### Data Analysis

We used Dedoose version 8.1.8 (University of California, Los Angeles) to manage the coding process. The first author (MR) read all interview transcripts and developed an initial codebook containing deductive codes about the extent to which caregivers perceived STAR-VTF to align with their informational, educational, psychosocial, and accessibility needs. Two members of the research team independently coded interview transcripts using the deductive codes in the initial codebook. They applied additional inductive codes based on the content of responses that were not covered by the original deductive codes. For all 15 transcripts, after coding each transcript, we reviewed the coding as a group. During these meetings, we reconciled coding disagreements through group discussion and transcript review. We revised and expanded the initial codebook throughout the coding process. After completing coding, members of the research team met regularly to discuss the coded excerpts; identify themes representing caregivers’ informational, educational, psychosocial, and accessibility needs; and identify exemplary quotes to represent each theme.

## Results

### Characteristics of Study Participants

From the EHR and claims data, we identified 54 potential study participants (ie, caregivers of patients who met the patient eligibility criteria) and mailed packets to them. Among these, 12 caregivers could not be reached by telephone, 17 were ineligible, and 8 declined participation. We scheduled interviews with the remaining 17 who were eligible and interested in participating. We were unable to conduct 1 interview because the patient with ADRD died before the scheduled interview. We were unable to conduct 1 second interview because the caregiver was not aware that the patient had an ADRD diagnosis, and the research team determined it would be difficult to have a meaningful conversation about the caregiver’s experience caring for a person with ADRD. Therefore, we completed interviews with 15 caregivers (Table 1).

In the following sections, we present the informational, educational, psychosocial, and accessibility needs that family caregivers perceived would prompt their use of a program such as STAR-VTF. In addition, we present characteristics of STAR-VTF that caregivers perceived would address their needs (Table 2).

**Table 1:** Characteristics of family caregivers and description of the caregiving situation (N=15).

Characteristic		Family caregivers
Age (years), mean (SD)		72 (10)
Female, n (%)		10 (67)
**Race, n (%)**		
	White	13 (87)
	American Indian or Alaska Native	1 (7)
	Asian	1 (7)
Hispanic or Latino ethnicity, n (%)		0 (0)
**Education, n (%)**		
	Post–high school training other than college (vocational or technical)	2 (13)
	Some college	4 (27)
	College graduate	8 (53)
	Postgraduate	1 (7)
**Occupational status, n (%)**		
	Employed	3 (20)
	Unemployed	1 (7)
	Student	1 (7)
	Retired	10 (67)
**Income (US $), n (%)**		
	$20,000 to $34,999	1 (7)
	$35,000 to $49,999	1 (7)
	$50,000 to $74,999	4 (27)
	$75,000 to $99,999	5 (30)
	$100,000 to $199,999	1 (7)
	$200,000 or more	1 (7)
	Prefer not to answer	2 (13)
Member of Kaiser Permanente Washington, n (%)		9 (60)
**Relationship to person with dementia, n (%)**		
	Spouse or partner	10 (67)
	Child	2 (13)
	Other family member	2 (13)
	Friend	1 (7)
Lives with person with dementia, n (%)		15 (100)
Duration in caregiving role (years), mean (SD)		5 (5)
**Caregiving hours per week, n (%)**		
	15 to 20	2 (13)
	21 to 24	1 (7)
	35 or more	12 (80)

**Table 2:** Summary of caregivers’ perceptions about needs and STAR-VTF characteristics that would address them.

Family caregiver perceived needs		STAR-VTF^a^ characteristics to address needs
**Information and education**		
	What BPSD^b^ to expect	Providing education earlier in disease stage (prior to symptoms) about what BPSD a person with dementia could experience
	Tailored help on how to manage BPSD	Offering examples of problem-solving strategies that could work for the particular BPSD caregiver is dealing with
**Psychosocial support**		
	Encouragement	Incorporating words of encouragement throughout training as caregiver learns to manage BPSD
	Coping with BPSD	Teaching caregivers strategies for managing their own frustrations with BPSD
	Supportive services	Connecting caregivers with vetted respite care and other supportive services, including caregiver support groups
**Accessibility**		
	Timing of program	Offering STAR-VTF to caregivers earlier in their roles as caregivers
	Modality of program delivery	Making STAR-VTF virtually accessible to provide convenient and timely help
	Time required to participate	Breaking up content into small chunks that caregivers could review as time and space permits
	Access to a designated provider	Having the same coach assigned to caregivers throughout duration of program

^a^STAR-VTF: STAR-Caregivers Virtual Training and Follow-up.

^b^BPSD: Behavioral and psychological symptoms of dementia.

### Information and Education Needs

The idea of the health care organization offering a virtual training program for caregivers to learn to manage BPSD was largely appealing to caregivers. They discussed the importance of learning early what BPSD they could expect in the person with dementia as the disease progressed and receiving tailored help on how to manage BPSD.

#### Learning Early What BPSD to Expect

When the person with dementia was diagnosed, caregivers were only vaguely aware of BPSD that some people with dementia experience. One caregiver expressed this scenario as “going in blind.” As the disease progressed, caregivers were startled to observe symptoms they did not anticipate, such as the person with dementia crying excessively. According to caregivers, health care providers did not provide sufficient warning of the personality and behavioral changes they might observe in the person with dementia. These changes became a source of deep frustration for caregivers, as they neither expected nor felt prepared to manage them. In response to the idea of STAR-VTF, one caregiver explained why it would be valuable to incorporate education earlier in the disease stage (prior to symptoms) about what BPSD a person with dementia could experience:

I can see where caregivers will just fall apart with some of these behaviors if you don't know what's coming. And it's not that I have anything against the doctors. They don't have time. Maybe they don't even know, but they don't have time to really prepare you for what you're taking on. But that's what caregivers need to be told from the beginning. Are you going to be caring for your husband or, you know, your parent? Here's a program to help you, to help you strategize through these difficult behaviors that might lie ahead for you. I think that really appeals to me and needs to be said in the very beginning, not when you're extremely desperate.

Caregivers also explained that knowing BPSD could occur and worsen as the disease progressed would help them to plan medical procedures (eg, elective surgery) before it became too challenging for the person with dementia to comply. In addition, it would help caregivers plan home modifications to ensure a safe environment for the person with dementia.

#### Receiving Tailored Help on How to Manage BPSD

After viewing prototypes of STAR-VTF, caregivers recognized the usefulness of learning problem-solving strategies that they could then apply to any challenging symptom. However, caregivers stressed their need to also receive tailored advice for problem-solving specific BPSD. For example, one caregiver suggested adding a search feature to STAR-VTF to find targeted advice on how to solve particular symptoms:

Right now, it's very general, okay? So, this is how you—this is what process you go through to solve issues. And the better you get at that process, the better you are at solving the issues, right? That come up in your day-to-day lives. But if you have a specific issue that is kind of quirky or way off in right field…Is there a backup situation where you can look something up? So that could be added on to this program. Once they [caregivers] go through the basic training.

A factor driving the suggestion to include tailored advice within STAR-VTF was caregivers’ desire to quickly address a challenging symptom. Some caregivers described feeling “desperate” when the person with dementia was experiencing BPSD. While caregivers recognized the value of going through the trial-and-error, problem-solving process that STAR-VTF teaches, they also perceived the amount of time required to go through this process as a limitation:

It's not something that, okay, we can spend the next week and a half going over this and trying...It's something—you need something, like, within an hour type of deal…that's what I found more often when we had serious concerns or questions, you know, I would go online and search other people having the same exact circumstances and how did they handle it and what worked and what didn't work.

In those difficult moments, caregivers desired to know specific strategies to try immediately to improve the situation. Thus, according to caregivers, STAR-VTF could be improved by incorporating a combination of teaching general problem solving and offering examples of strategies that could work for particular BPSD as they arise.

### Psychosocial Support Needs

Caregivers discussed their psychosocial needs and how they envisioned STAR-VTF to support them. In particular, caregivers described a need to receive encouragement, learn how to cope with BPSD, and connect with supportive services.

#### Encouragement When Learning to Manage BPSD

Through educational materials, family members and friends, other family caregivers of people with dementia, and their own experience, some caregivers had identified successful strategies for managing particular BPSD. For example, at the suggestion of her daughter, one caregiver used a whiteboard to write answers to questions the person with dementia asked repeatedly so that the caregiver could point to the whiteboard instead of having to constantly repeat herself. Nonetheless, the process of identifying successful strategies was hard. They were discouraged when strategies that worked for other caregivers in managing a particular symptom did not work for them. They were also discouraged when strategies that had worked for them in the past were no longer effective. Consequently, caregivers underscored the importance of STAR-VTF offering encouragement during the process of learning to manage challenging symptoms:

Caregivers carry around a lot of guilt because they want to be perfect. We love our mates. We love whoever we're caring for. We want to do it right. What worked last week doesn't necessarily work this week. So, if a solution that worked last week doesn't work, then persevere. I would say that. Persevere for another solution…Caregivers need to be assured that no one solution is going to be perfect for everyone. But to persevere, because there is something out there that will work.

Assuring caregivers that what worked for one isn't necessarily going to work for another. Because as a caregiver, you so often have people say to you, “oh, yeah, well, so-and-so did this and had no problem”...So, I guess caregivers need to be assured that if something doesn't work, it doesn't mean that they are a failure; it just means try—here are some other things to do.

Caregivers expressed differences in opinion on how this type of encouragement could be embedded within STAR-VTF. Some suggested the STAR-VTF self-guided modules could display words of encouragement throughout the training and tailor these messages based on caregivers’ responses to interactive exercises. Others felt that only a human could express this empathy. One caregiver stated:

It's that personal relationship of somebody understanding where you are and, you know, patting you on the shoulder and saying that must be really tough. And I don't know how you convey that the same way on a website.

#### Coping With BPSD

Symptoms of people with dementia that caregivers described as particularly challenging were insomnia, asking repetitive questions, and having trouble getting to the toilet. Caregivers described their frustration with not understanding why the BPSD were happening and how they could handle them. When asked how they envisioned STAR-VTF would provide support, a theme that resonated with many caregivers was teaching strategies for managing their own frustrations with BPSD. One caregiver suggested that STAR-VTF put more emphasis on reducing the caregiver’s frustration rather than reducing the challenging symptom in the person with dementia:

So, you said something about helping reduce the challenging behaviors? If you phrase it like that, it makes it sound like that's something that can be fixed. And maybe then what happens if it doesn't change? I just, you know, maybe the emphasis could be on how you deal with the challenges in a way that's less frustrating for both you and the patient as opposed to reducing the challenging behavior.

According to caregivers, health care providers treating the person with dementia did not offer support to help caregivers cope with the frustration of BPSD. Caregivers wanted health care providers to acknowledge their challenging situation and direct them to supportive services. One caregiver said that for 4 months, a neurologist was trying different medications to treat her husband’s insomnia and other behavior symptoms. Of this time, she stated:

Those are four months where I needed someone, and I didn't have anybody to talk to. And I think if caregivers, for instance, were told by their doctors—and we are part of Kaiser—it would be wonderful to have them say there's help for you. I needed someone to tell me this is a very challenging job. And it's not that I wasn't aware. Because I have a brother who took care of his wife with Alzheimer's, and, you know, I knew all that, but I needed a doctor to tell me this is really difficult. There's help for you. And there's this program

STAR-VTF

What motivated caregivers to improve their own mental health and well-being was a perception that if they felt better emotionally, they would be better able to provide care to the person with dementia. As one caregiver put it:

My goal is to keep her here at home as best I can. And if I break down, then you've got two to take care of.

#### Supportive Services

Throughout the different stages of dementia, caregivers reported taking it upon themselves to seek education and supportive services. In the early stages, caregivers described a desire to gain a better understanding of dementia and its progression. One caregiver described how invaluable it had been for her to read books and academic articles on the topic. After viewing prototypes of STAR-VTF, the caregiver suggested adding more content that explained what dementia is and providing additional reading materials:

I thought that there ought to be definitions and some sort of information on what dementia is and Alzheimer's is, the differences. Not a great deal of difference, but just a—that sort of thing for me would be important. Books to read, you know.

In the middle and later stages of the disease, caregivers described a need to learn what respite services were available to provide short-term breaks from caregiving. Caregivers expressed an interest in both in-home services and adult day care programs. However, according to caregivers, finding trustworthy service providers was difficult. Some caregivers talked about negative experiences with service providers they had identified online. Caregivers strongly desired for the health care organization to vet respite care programs and guide caregivers on how to choose a program that was right for their unique situation. One caregiver suggested that the role of the STAR-VTF coach include connecting caregivers with respite care and other supportive services:

I know one of my things coming up is that I'm going to need to have in-home assistance, and it's for short term—maybe it's just a daycare that I need to take him to but where are they, who they are, if we could have resources that we could look at. Thinking this will meet this need, this will meet that need, and at least give you someone [in STAR-VTF] to access and say, “Hi, I'm so-and-so and this is my situation. Can you help me?”

Throughout their caregiving journey, caregivers described a desire to learn about and join a caregiver support group. Caregivers who had joined a support group stated that it was helpful to share their stories with other caregivers and get advice on various aspects of caregiving. They were interested in the health care organization helping caregivers to connect with support groups for family caregivers of people with dementia. For example, one caregiver described an online support group she participated in regularly and suggested that STAR-VTF inform caregivers about it:

I've been in touch with an online group…that is designed for people who are dealing with caregiving of those with Alzheimer's. And they allow you to post any kind of question you have, any kind of circumstance you found yourself in, and see what kind of feedback others can give you. And it's really an excellent resource…Sharing information of what they have experienced and how they dealt with it to give you ideas…And so, with your program [STAR-VTF], I don't know if you have a place where you're going to link to other possible resources. This [online caregiver support group] would be a strong resource to link to.

The internet was a major source of information for most caregivers. However, they often found the amount of information online to be overwhelming. Furthermore, despite the vast amount of information online from different types of sources, caregivers desired a single source (“one-stop shop”) of comprehensive and high-quality information. To that end, caregivers envisioned that STAR-VTF would consolidate educational materials and information on the availability of supportive services. Caregivers could then rely on and turn regularly to STAR-VTF as a major source of information, knowing that it came from the health care organization, which caregivers unanimously perceived as trustworthy.

### Accessibility Needs

Caregivers discussed needs related to the accessibility of STAR-VTF, including the timing of offering the program to caregivers, modality of program delivery, time required to participate, and inclusion of a designated provider.

#### Timing of Program

When asked whether they could envision using STAR-VTF, caregivers answered affirmatively but expressed a desire for the program to be offered earlier in their role as caregivers for multiple reasons. As described above, caregivers suggested providing information about BPSD before the person with dementia begins experiencing symptoms and before caregivers begin trying their own strategies for challenging symptoms. These caregivers perceived that STAR-VTF would have been more useful to them when symptoms first began appearing:

This [STAR-VTF] would be key to get into people's hands at the earliest possible time. Because beyond that, then somebody—I mean, like we've already—we learned by trial and error how to do exactly what you're saying to do in this program.

Having this [STAR-VTF] as something to help you adjust early on and not have to learn on your own is definitely a plus…because we have already kind of figured out some plans and realized certain things. And our own private research got us to where we are, you know. And, of course, I mean, obviously, we talked to docs a few times, but we were already on a pathway of controlling behaviors, in other words. When she got into stage 3, we had a pretty good handle on things by then—but this [STAR-VTF] would have definitely helped when we first got her five years ago.

Another reason why caregivers suggested that STAR-VTF be offered earlier is so the STAR-VTF coach could be with them from the start. According to caregivers, the ideal scenario would be for the coach to get to know each caregiver and their caregiving situation over time. Caregivers could then trust the coach’s advice, knowing that it was informed by an in-depth understanding of their unique situation. One caregiver indicated that she would be reluctant to trust a coach whom she had met only in the later stages of her husband’s disease:

If I'm really going to put my trust in this program, I would probably have to start at the beginning. I don't think if I jumped in in the middle or towards the end, I would feel like, well, they [the coach] didn't know about this earlier on, and so maybe the information they're giving me now isn't considering what happened. But I think if you probably started right from the start, like he was diagnosed today and so you said go onto this online program and we could help you, I think you could grow with the program and I think develop a pretty good trust.

Thus, while the reasons that drove preferences for the timing of the program differed among caregivers, the consensus was that STAR-VTF would be a valuable support if it were offered early in their caregiving experience.

#### Modality of Program Delivery

The virtual aspect of STAR-VTF was appealing to the majority of caregivers, including those who did not consider themselves to be technologically savvy. The latter group underscored their need for a user-friendly design, particularly so they could easily locate and navigate the STAR-VTF website, recover from errors, adjust the text size, and receive technical assistance. Caregivers perceived that participating in a virtual program from home would be more convenient than a program requiring in-person attendance at a health care facility. According to caregivers, the latter would necessitate finding someone to care for the person with dementia or having the person with dementia accompany them to the in-person visit. For some caregivers, neither of these options would be practical. Hiring professional care would be costly and taking the person with dementia to a health care facility would be challenging. For one caregiver, virtual participation was the most attractive aspect of STAR-VTF, given the difficulty of getting her mother to accompany her on an in-person visit:

That's one of the problems we have is it's almost impossible to get her out of the house, you know, she gets so upset, you know, going to the doctor…So, I can see—that's where I would see the most important part about it [STAR-VTF] being online is you can get that help at home.

There was also a perception among caregivers that a virtual program would result in more timely help compared with the delays they sometimes experienced when seeking help from the health care team through traditional modalities. As one caregiver described, as long as a device was available to access the STAR-VTF website, caregivers would have help at their fingertips. She went on to say:

You wouldn’t have to call a doctor to get answers…It's there when you're having those feelings and they're very frustrating feelings and you don't know what to do…If you're at the end of your rope and you don't have an answer, you can immediately sit down at your computer or your tablet or with your phone and get in there and it's like, I'm frustrated. Let's see why. Yeah, that's the behavior that he's doing. It affects me this way. Let's figure out what we can do about it. It's having this—it's right there. It's here at the house. It's not, you know, something that I have to make—I spend so much time on the phone waiting…over 35 minutes on the phone waiting to speak to the neurologist's nurse the other day.

Caregivers hoped that STAR-VTF would enable them to access help the moment they experienced a need instead of having to wait to speak to a member of the health care team on the phone or in person. (Some caregivers reported a 2- to 3-month wait time for a clinic visit with a specialist.) All caregivers owned at least one device (eg, smartphone, tablet, laptop, desktop computer) they could use to participate in a virtual training program.

#### Time Required to Participate

Even with a virtual program, caregivers were concerned about the limited amount of time and space they had to devote to STAR-VTF. The vast majority of study participants provided care to the person with dementia for 35 hours or more per week (Table 1). Caregivers said the person with dementia would likely make it difficult for them to concentrate for long stretches of time on the STAR-VTF training materials. When asked to elaborate, caregivers used expressions such as “needy,” “constantly interrupts me about things,” and “requires a lot of attention” to describe the person with dementia. One caregiver explained what she would need to be able to fully engage with the STAR-VTF online content:

I would need privacy and to be away. I couldn't be in the same room with my husband [with dementia] who was giving me grief or being demanding or unpleasant or whatever. So, I think for me to use the program, I would have to be in a place where I could presume I have some privacy and some time, and it requires the concentration to be able to focus on it and not being distracted by other things, I think.

To facilitate their use of STAR-VTF under these circumstances, caregivers suggested that the online content be broken up into small chunks that they could review as time and space permitted. Caregivers also suggested having the ability to pause a module, if needed, and then be able to pick up where they left off when they returned to it later.

#### Having Access to a Designated Provider

Of the aspects of STAR-VTF that we described using prototypes, of notable interest to caregivers was the availability of a coach to provide support beyond the self-directed online materials. Caregivers described an unmet need to have access to a designated health care professional they could turn to for help when they were experiencing difficulties with BPSD. According to caregivers, people with dementia receive care from multiple health care providers within the same practice area, depending on appointment availability. For example, one caregiver described how his wife with dementia had recently visited 3 different primary care providers, all at different health care facilities. While these providers were, “real nice, really good, and really helpful,” the caregiver desired a single provider who had direct knowledge of their clinical and caregiving situation instead of a provider who “doesn’t have any idea except what they see in the medical record.” Thus, if caregivers were to participate in STAR-VTF, it mattered considerably that the same coach be assigned to them throughout the duration of the program. Caregivers described being in extremely stressful situations that sometimes resulted in them losing their temper, becoming angry, and yelling at the person with dementia. These would be the types of situations caregivers would want to share with the STAR-VTF coach, simply to vent but also to receive help.

However, caregivers need to trust the coach to feel comfortable sharing openly and honestly about the caregiving situation. Caregivers explained that trust would develop only with time and repeated interactions with the same person. In response to a question about whether caregivers would be willing to share with the coach their responses to interactive exercises within the self-directed online materials, one answered yes but only if it were consistently the same coach with whom they had a trusting relationship:

Yes. Here we're talking about a coach that I've been working with on and on and on because I'm working on the program, right? So, I have developed trust with the coach. So, yes, I would talk to her about that. But, I would be a little hesitant if the coach was brand new, I didn't know her, I didn't have any experience with her, and then maybe one week this one comes, but next week she can't come so a substitute comes and, I mean, I know they're both qualified, but you don't know the second person and you don't know whether the trust is there as much as with the first person.

In addition to having access to a designated STAR-VTF coach, caregivers discussed a desire for this person to have extensive practice experience working with people with dementia and their family caregivers. This qualification would help caregivers trust the information provided by the coach.

## Discussion

### Principal Findings

We found that family caregivers of people with dementia were interested in the idea of a virtual training program for learning to manage BPSD. Caregivers in our study reported that health care providers did not provide adequate education in the early stages of the disease about the types of personality and behavioral symptoms that can affect people with dementia. When the person with dementia began experiencing BPSD, it was unexpected and frustrating for caregivers, since they felt unprepared to manage the symptoms. For this reason, caregivers expressed a strong desire for the health care organization to offer programs such as STAR-VTF much sooner. When we interviewed them, many caregivers were reflecting on their extensive experience with BPSD, leading them to recommend STAR-VTF for those with less experience.

Furthermore, the virtual aspect of the training program appealed to nearly all caregivers in our study. Caregivers were interested in virtual training because of the convenience of receiving help from home and a perception that a virtual program would result in more timely help compared with traditional service modalities (eg, face-to-face visits, calling consulting nurse service). Given caregivers’ limited time and privacy for reviewing the STAR-VTF training materials, they suggested breaking up the content into small chunks that they could review as time and space permitted. Finally, caregivers desired continuity by having the same STAR-VTF coach assigned to them throughout the program duration. Collectively, our findings provide a better understanding of the type of support that caregivers need to manage BPSD. Our results indicate that caregivers perceived that features of STAR-VTF could address their needs.

Based on our findings, which were collected during the intervention design process, we improved STAR-VTF for testing in our pragmatic randomized trial [10]. We incorporated a feature in the STAR-VTF modules that enables caregivers to pause the training and continue later from that point. Multiple members of the research team iteratively reviewed the modules in a testing environment and identified usability issues to address prior to trial recruitment. During the trial, we will collect and monitor structured responses from caregivers after their completion of each module to assess perceived usability and usefulness. Furthermore, we gave each STAR-VTF coach a designated panel of caregivers to promote continuity of support. STAR-VTF coaches were trained on the importance of expressing empathy and offering encouragement during difficult times, helping caregivers to problem solve specific behavioral symptoms, referring caregivers to KPWA community resource specialists to help them find supportive services (eg, respite care), and helping caregivers learn how to use their electronic devices to access the STAR-VTF training materials. We were unable to address caregivers’ preference to offer STAR-VTF earlier. Since caregiver outcomes are not tracked within the EHR, we currently have no pragmatic alternatives to using a prescription for an antipsychotic medication as a signal for when a caregiver needs help managing behavioral symptoms and could benefit from participating in STAR-VTF. New research is needed on earlier identification of caregivers who are experiencing problem behaviors in people with ADRD.

### Comparison With Previous Work

While family caregivers may not provide much assistance to people with dementia in the early stages of disease, Whitlatch and Orsulic-Jeras [14] argue that this is a critical time for family caregivers to obtain information and education about the disease, symptoms, and progression. In our study, caregivers believed they would have benefited from education about BPSD much earlier. This finding is consistent with research by Boots et al [15], which reported that retrospectively, family caregivers of patients with late-stage disease did not believe they had sufficient knowledge in the early stages about the manifestations of dementia. Furthermore, while health care providers are an important and trusted source of information for family caregivers of people with dementia [16], our participants reported that they did not provide sufficient information about the personality and behavior changes that people with dementia can experience. Complementary to our findings, Peterson et al [17] reported that the perception among caregivers was that health care providers offer little to no useful information about dementia and caregiving. In surveys, caregivers also report that their need for information on what to expect as dementia progresses is not met by health care providers [18,19].

STAR-VTF could be offered earlier, before the person with dementia begins experiencing changes in their behavior, as caregivers in our study suggested. For example, caregivers could learn about the activators-behaviors-consequences problem-solving strategy in advance before they need to apply it. Wald et al [20] observed that family caregivers of people with dementia requested that information about a variety of topics (including BPSD and its management) be provided at the time of diagnosis rather than when the need arose. However, in a study examining the acceptability of structured discussions about future care during the early stages of dementia, Orsulic-Jeras et al [21] found that dyads of a family caregiver and person with dementia perceived one of the major drawbacks of the program was discussing topics that did not apply to their current situation. For example, study participants stated that they did not currently have “those conditions” and were not currently experiencing problems. Thus, a compromise for STAR-VTF timing could be to first offer caregivers program components that are most relevant in the early stages of the disease (eg, realistic expectations about dementia and possible BPSD) and reserve components that are narrowly focused on problem solving for later stages when the person with dementia begins experiencing BPSD. How health care organizations can identify the optimal point at which caregiver needs for BPSD management begin to arise and thus offer the problem-solving components of STAR-VTF is a critical topic requiring future research. An important consideration is not offering the program too early, when caregivers deem the training irrelevant, but not waiting until the situation has escalated to the point of requiring antipsychotic medications.

The COVID-19 pandemic has substantially increased interest in virtual health services. We conducted interviews in the summer and fall of 2019, prior to the pandemic and the rapid shift to virtual services. At that time, most caregivers in our study perceived the virtual aspect of STAR-VTF as an attractive feature. After viewing low-fidelity protypes of STAR-VTF, caregivers noted the potential convenience of accessing support virtually and receiving timely help. We note that our study sample was predominantly White and highly educated, representing US groups with the highest internet usage. Of White adults, 92% use the internet (compared to 85% and 86% of Black and Latino adults, respectively), and 98% of college-educated adults use the internet (compared to 71% of adults with less than a high school education) [22]. All caregivers in our study had internet access at home and owned devices (eg, laptop, desktop) that would be required for participation in a virtual training program. A systematic review of internet-based interventions to support family caregivers of people with dementia reports that, among family caregivers with high digital literacy and internet access, such interventions produce beneficial impacts on caregiver depression, anxiety, and burden [23].

### Limitations

Our study has limitations worth noting. First, we used a dispensed prescription for an antipsychotic medication as a proxy for identifying family caregivers dealing with BPSD in the person with dementia. This inclusion criterion likely biased our study sample toward caregivers caring for a person in the later stages of dementia when BPSD are most prevalent. Their needs for help in managing BPSD and their perception of the potential usefulness of STAR-VTF could differ from caregivers who are caring for a person in the earlier stages of dementia. However, the experiences of family caregivers in our study made them especially knowledgeable about family caregiver needs regarding BPSD management throughout the disease trajectory. Second, our sample was of KPWA members living in 3 western Washington counties that included the Seattle metropolitan area. As a qualitative study, we did not aim for generalizability [24]; however, we note that our findings represent the perspectives of a limited group. KPWA members are predominantly White and highly educated, so 13 of 15 (87%) participants were White and none were Latino. Our experience suggests that Latino KPWA members may be less likely than non-Latino White members to be prescribed antipsychotic medications. Future research needs to explore this hypothesis.

### Conclusions

Our findings contribute new knowledge about family caregivers’ views on participating in a virtual training program for the management of BPSD. Family caregivers needed information about BPSD and help in managing it, and they stated that STAR-VTF had the potential to directly address these needs. Furthermore, caregivers were attracted to the convenience of accessing the training virtually. They felt that a virtual training program would be more beneficial if it were offered early in their caregiving experience. Accordingly, our findings shed light on the need for future research to identify the optimal point at which to offer STAR-VTF. Offering the program too early risks providing training that is irrelevant to caregivers’ current situation, while offering it too late risks providing training after caregivers have already spent significant effort problem solving challenging behaviors on their own. Overall, our findings provide evidence that family caregivers would likely accept a program such as STAR-VTF focused on BPSD management that is offered entirely virtually.

## Appendix

Multimedia Appendix 1 Storyboards used during interviews with caregivers.

Multimedia Appendix 2 An example of a low-fidelity prototype used during interviews with caregivers.

## Abbreviations

ADRD: Alzheimer disease and related dementias
BPSD: behavioral and psychological symptoms of dementia
EHR: electronic health record
ICD-9-CM: International Classification of Diseases Ninth Revision—Clinical Modification
KPWA: Kaiser Permanente Washington
STAR-C: STAR-Caregivers
STAR-VTF: STAR-Caregivers Virtual Training and Follow-up
