# Supplementary material for: Family Caregiver Needs and Preferences for Virtual Training to Manage Behavioral and Psychological Symptoms of Dementia: Interview Study
Source: JMIR Aging. 2021 Feb 10;4(1):e24965. doi: 10.2196/24965 (PMC8081155; doi:10.2196/24965)
Supplement: Multimedia Appendix 1 [file aging_v4i1e24965_app1.pdf]

Storyboard I: Caregiver Discovers STAR-VTF

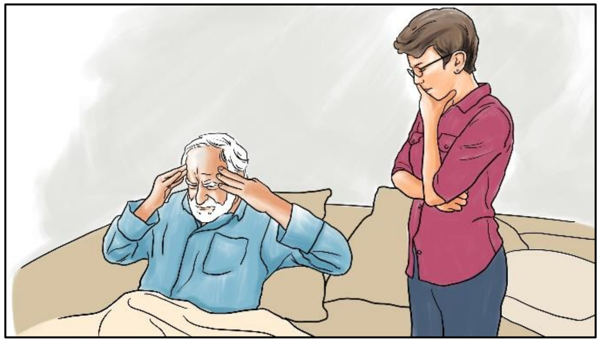

Caregiver does not know how to respond to person with dementia challenging behaviors

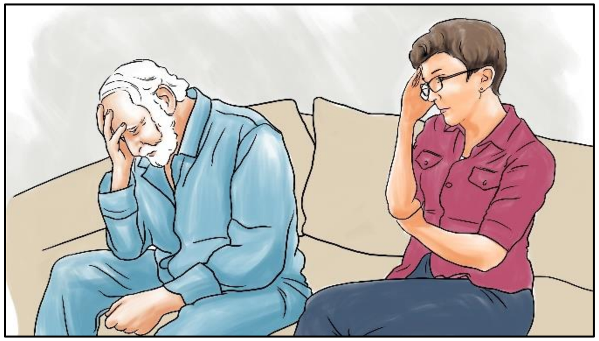

Caregiver and person with dementia both feel overwhelmed by the situation

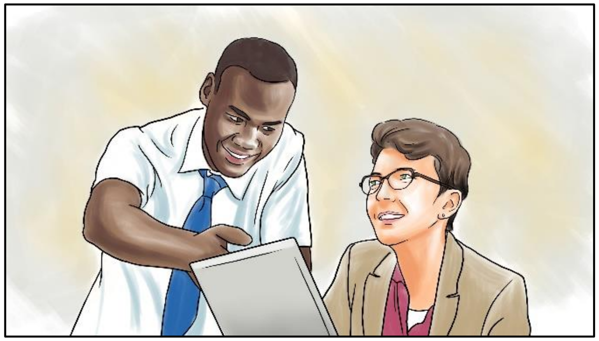

Caregiver talks to person with dementia clinical team and learns about STAR-VTF

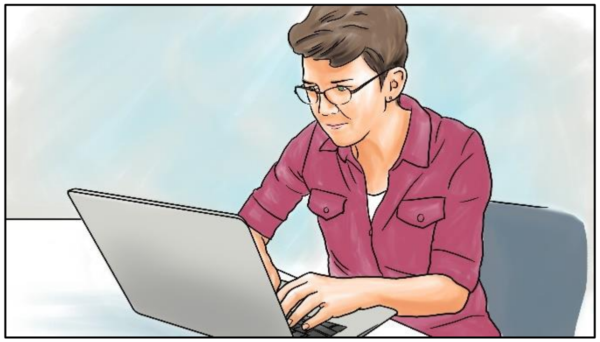

Caregiver uses STAR-VTF at home

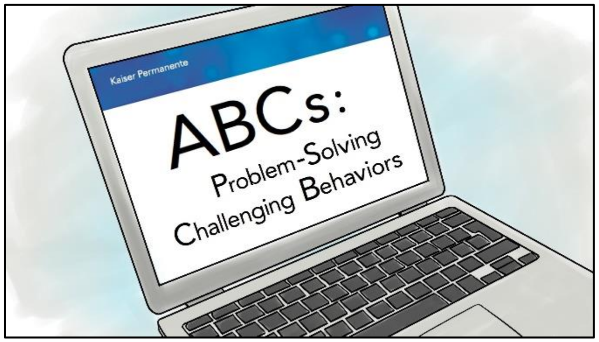

Caregiver learns strategies for managing person with dementia challenging behaviors

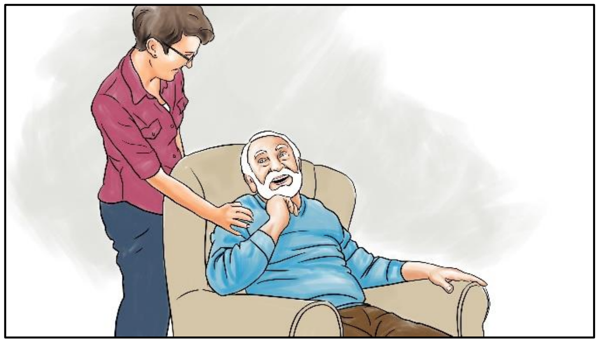

Caregiver and care recipient can better manage challenging behaviors and both feel calmer as a result

Storyboard II: Caregiver Uses STAR-VTF Program

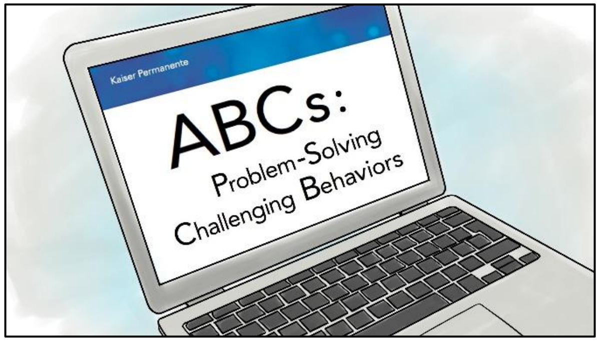

Caregiver learns that challenging behaviors are common, but there are ways to reduce them

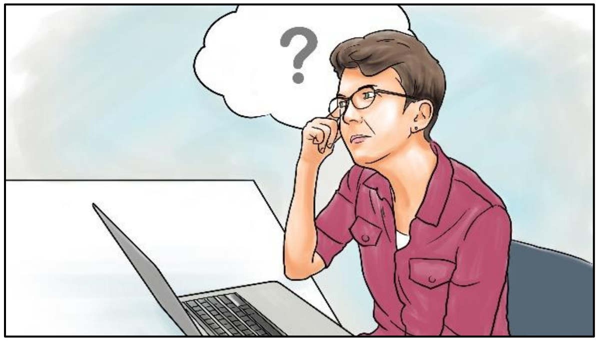

Caregiver chooses which behavior to focus on

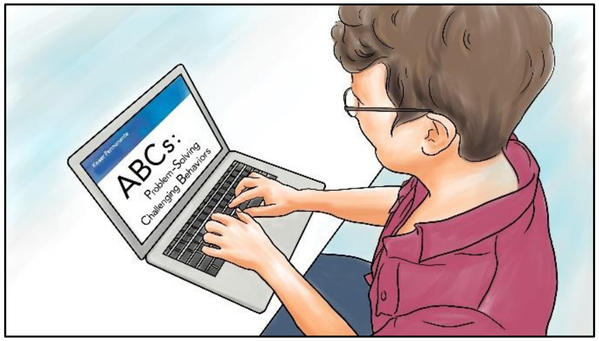

Caregiver creates a strategy for how to respond to this behavior

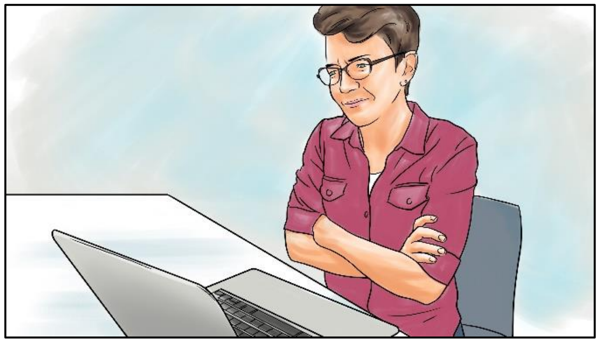

After reviewing material, caregiver has unanswered questions

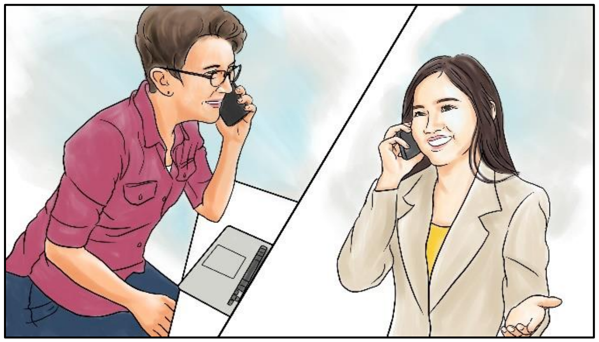

Caregiver sends an email or calls a coach for help

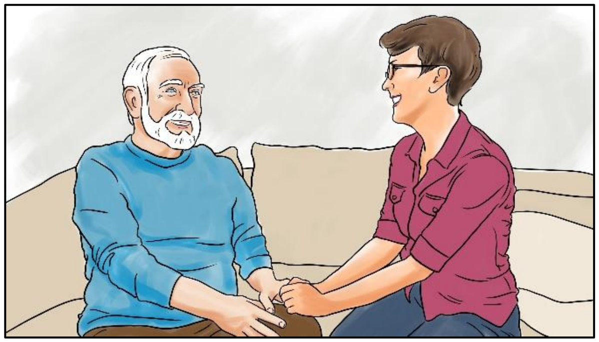

Caregiver uses strategy and it helps with the challenging behavior
