# Supplementary material for: Family Caregiver Needs and Preferences for Virtual Training to Manage Behavioral and Psychological Symptoms of Dementia: Interview Study
Source: JMIR Aging. 2021 Feb 10;4(1):e24965. doi: 10.2196/24965 (PMC8081155; doi:10.2196/24965)
Supplement: Multimedia Appendix 2 [file aging_v4i1e24965_app2.pptx]

## Slide 1
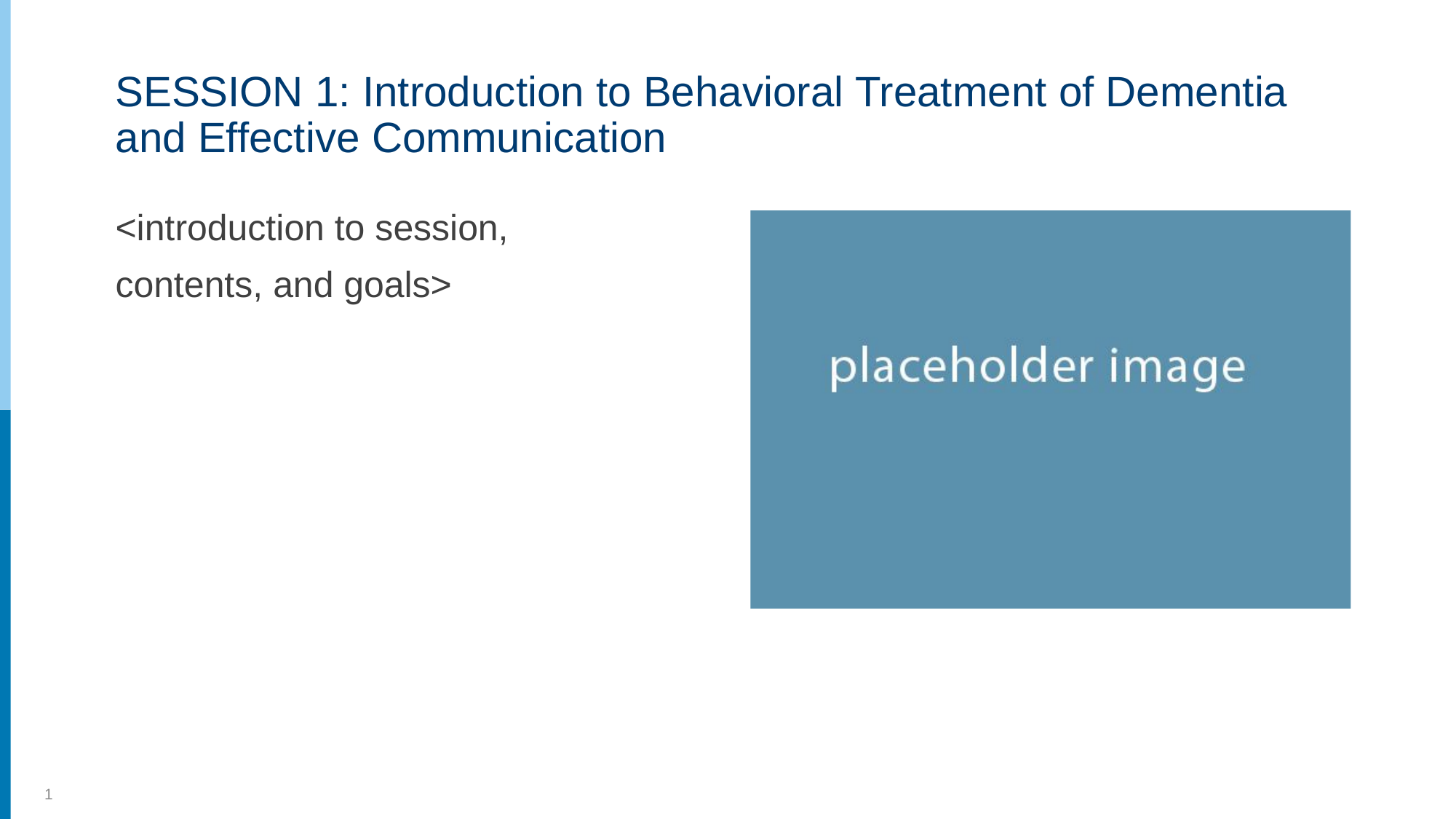

SESSION 1: Introduction to Behavioral Treatment of Dementia and Effective Communication
<introduction to session, contents, and goals>

## Slide 2
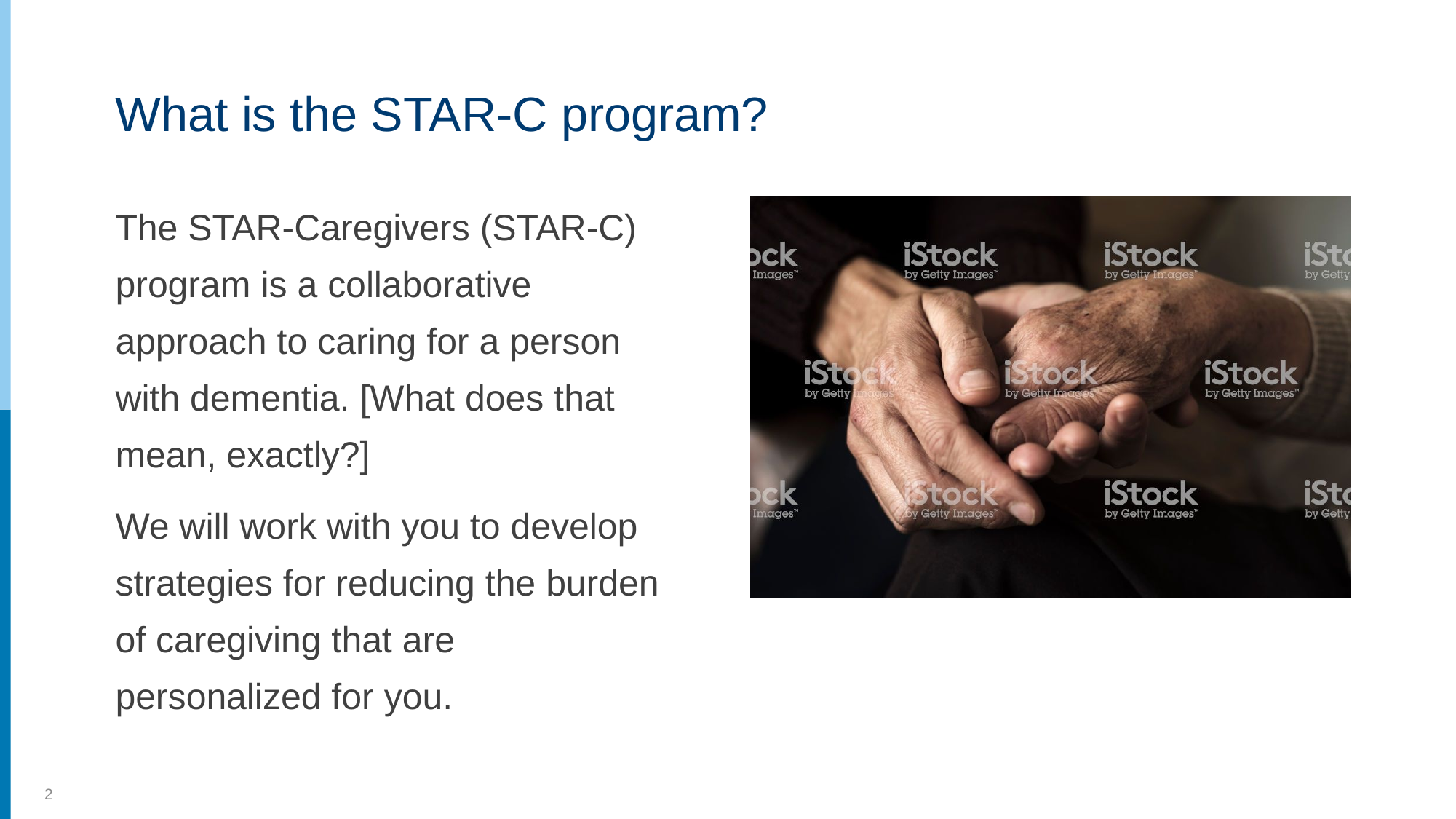

What is the STAR-C program?
The STAR-Caregivers (STAR-C) program is a collaborative approach to caring for a person with dementia. [What does that mean, exactly?]
We will work with you to develop strategies for reducing the burden of caregiving that are personalized for you.

## Slide 3
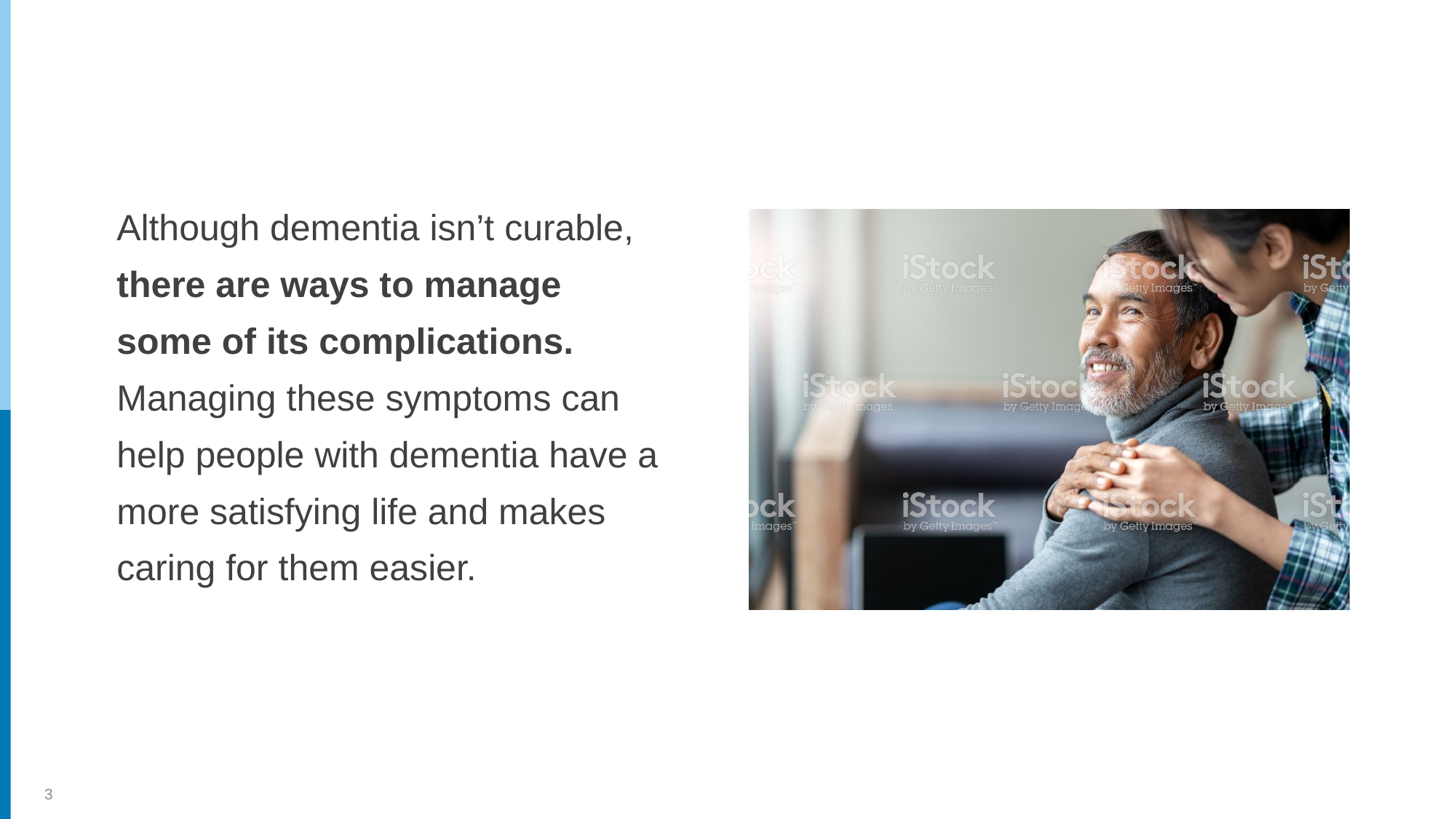

Although dementia isn’t curable, there are ways to manage some of its complications. Managing these symptoms can help people with dementia have a more satisfying life and makes caring for them easier.

## Slide 4
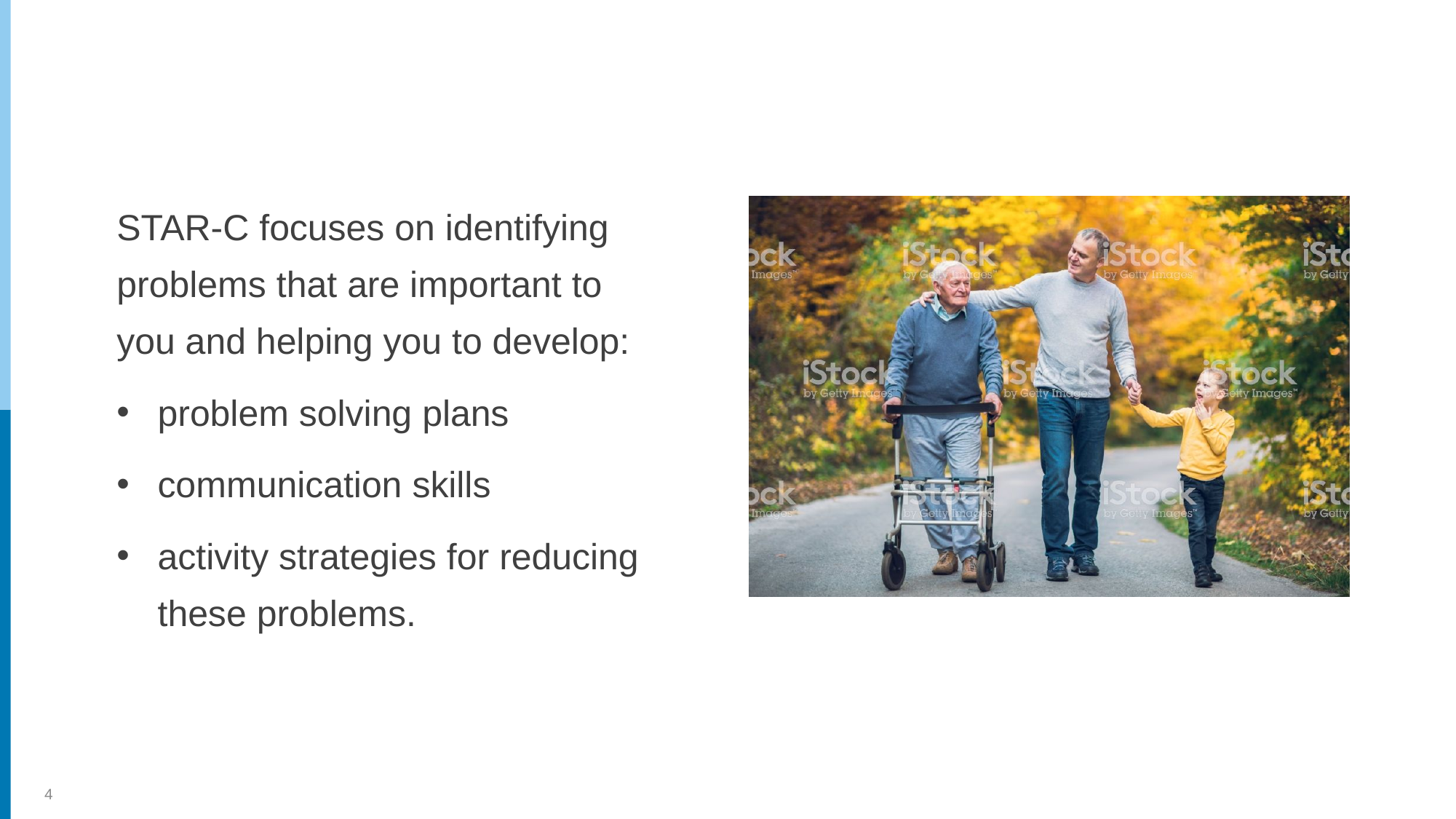

STAR-C focuses on identifying problems that are important to you and helping you to develop:
problem solving plans
communication skills
activity strategies for reducing these problems.

## Slide 5
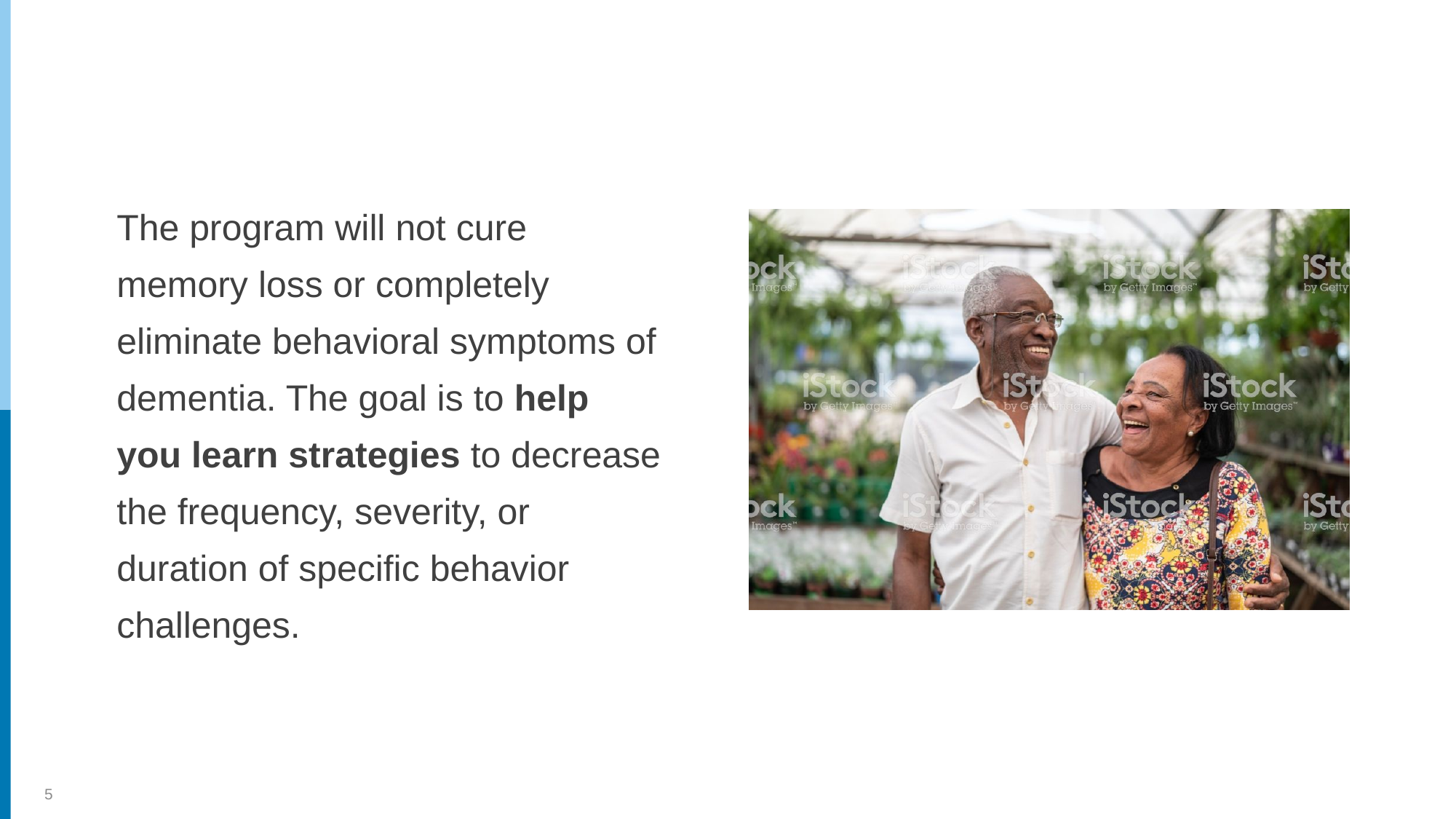

The program will not cure memory loss or completely eliminate behavioral symptoms of dementia. The goal is to help you learn strategies to decrease the frequency, severity, or duration of specific behavior challenges.

## Slide 6
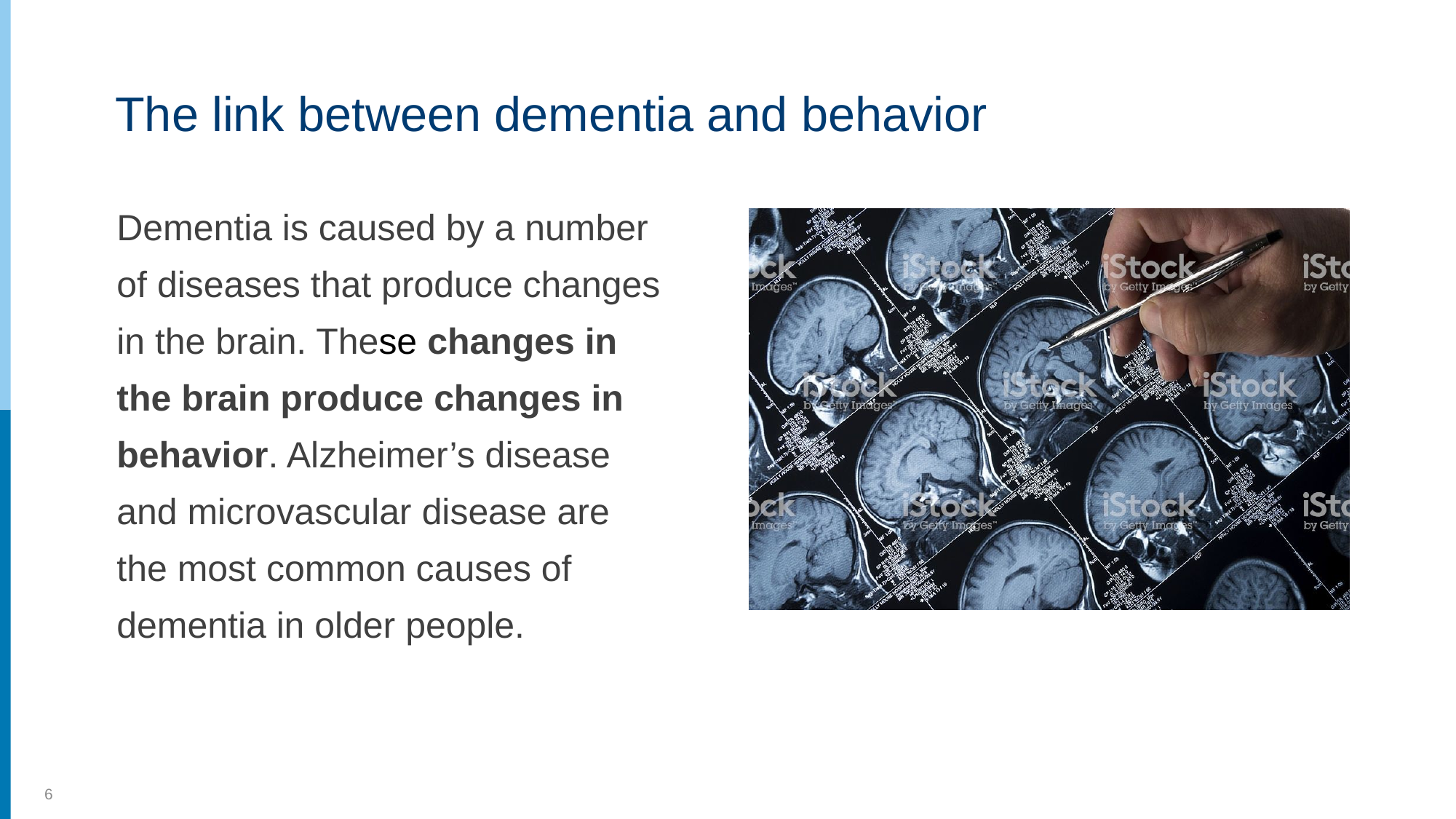

The link between dementia and behavior
Dementia is caused by a number of diseases that produce changes in the brain. These changes in the brain produce changes in behavior. Alzheimer’s disease and microvascular disease are the most common causes of dementia in older people.

## Slide 7
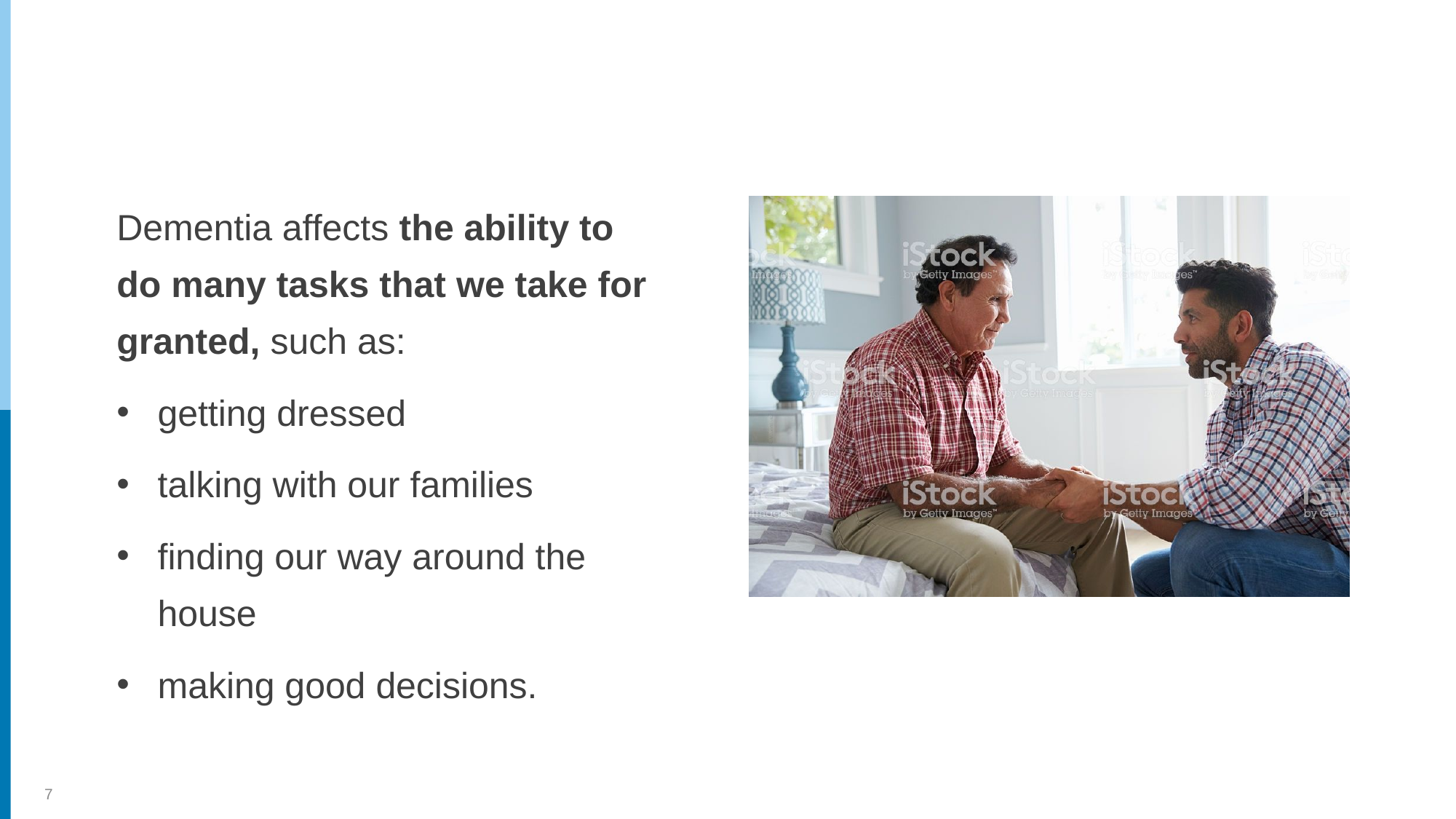

Dementia affects the ability to do many tasks that we take for granted, such as:
getting dressed
talking with our families
finding our way around the house
making good decisions.

## Slide 8
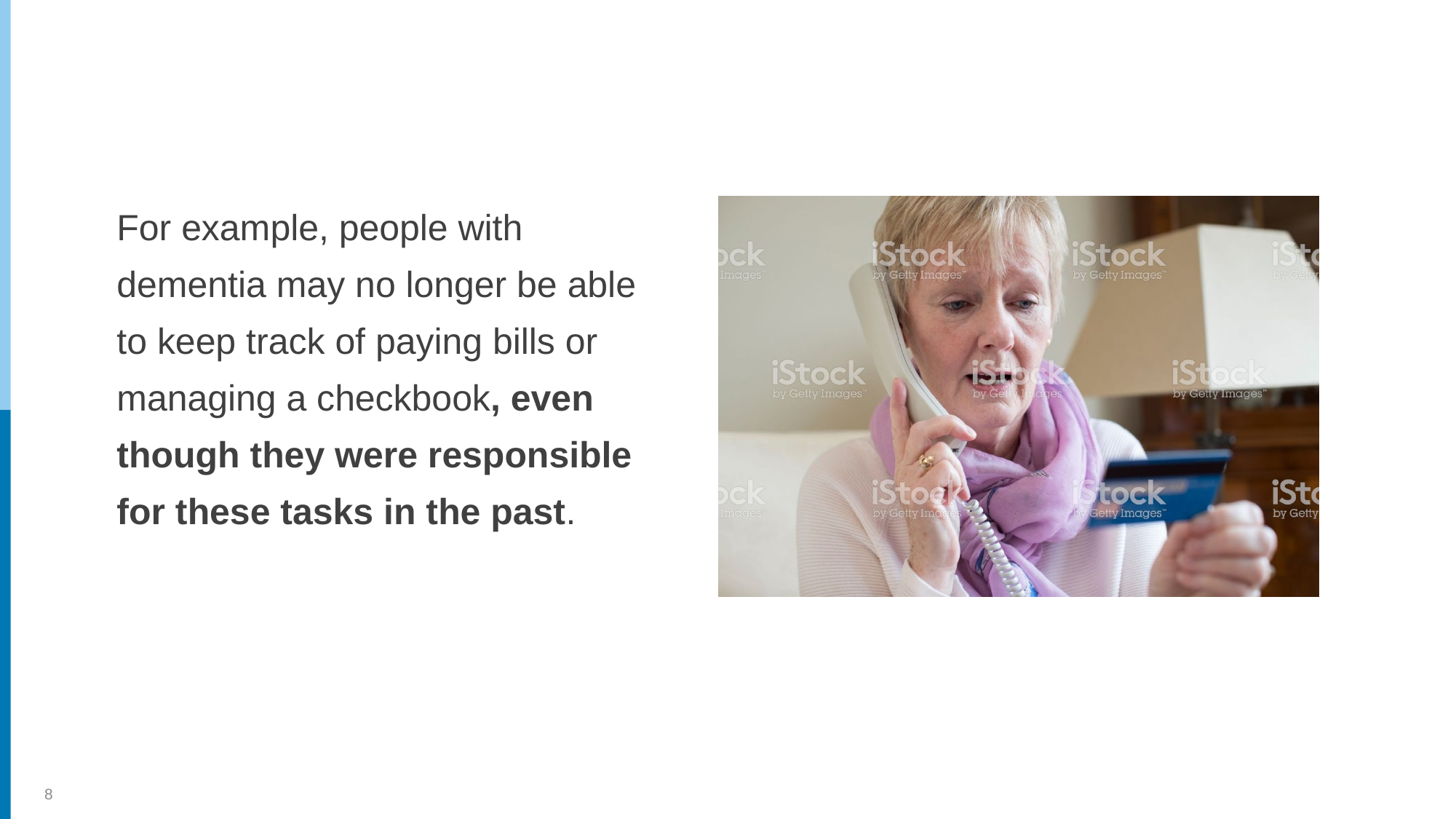

For example, people with dementia may no longer be able to keep track of paying bills or managing a checkbook, even though they were responsible for these tasks in the past.

## Slide 9
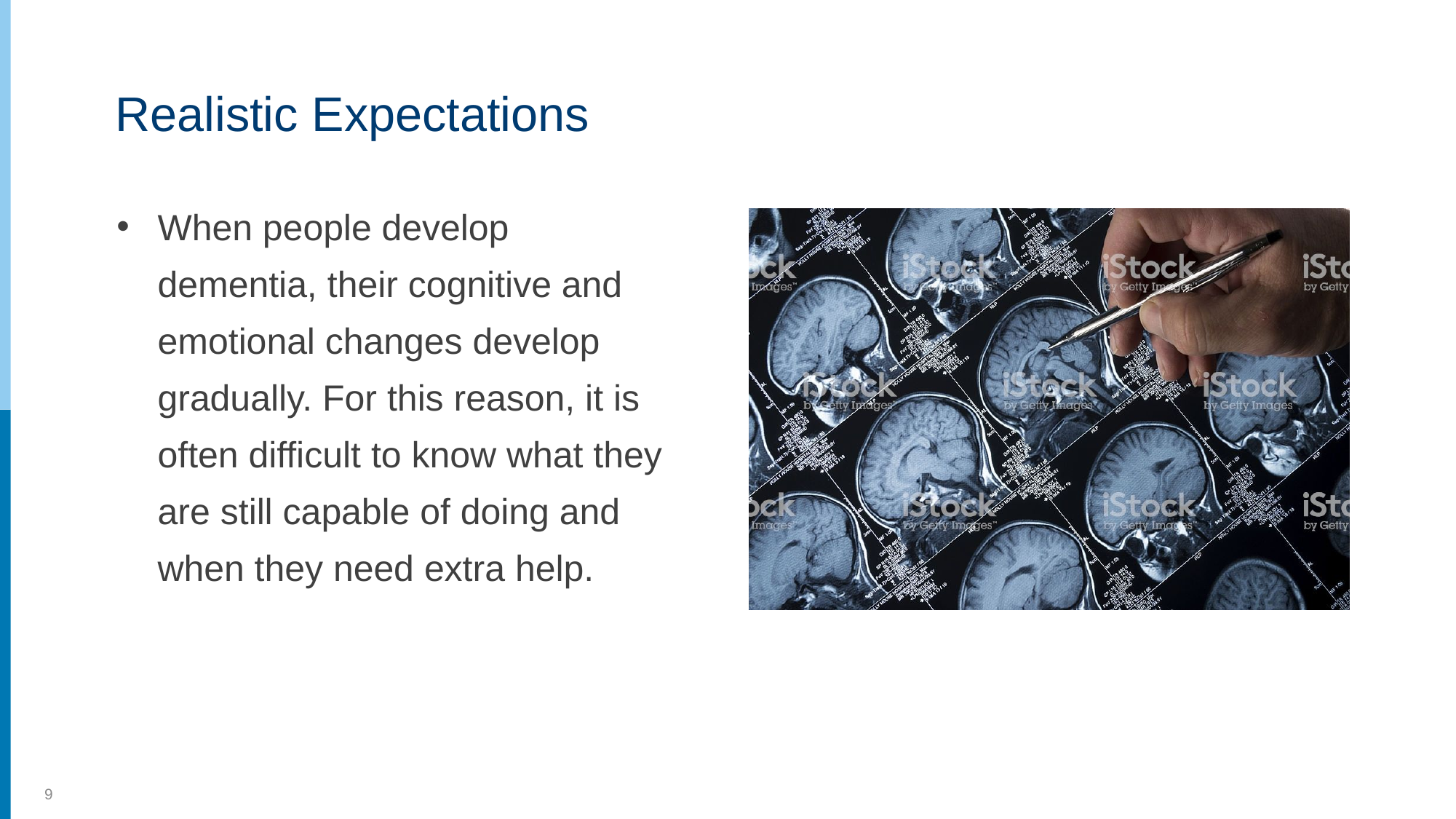

Realistic Expectations
When people develop dementia, their cognitive and emotional changes develop gradually. For this reason, it is often difficult to know what they are still capable of doing and when they need extra help.

## Slide 10
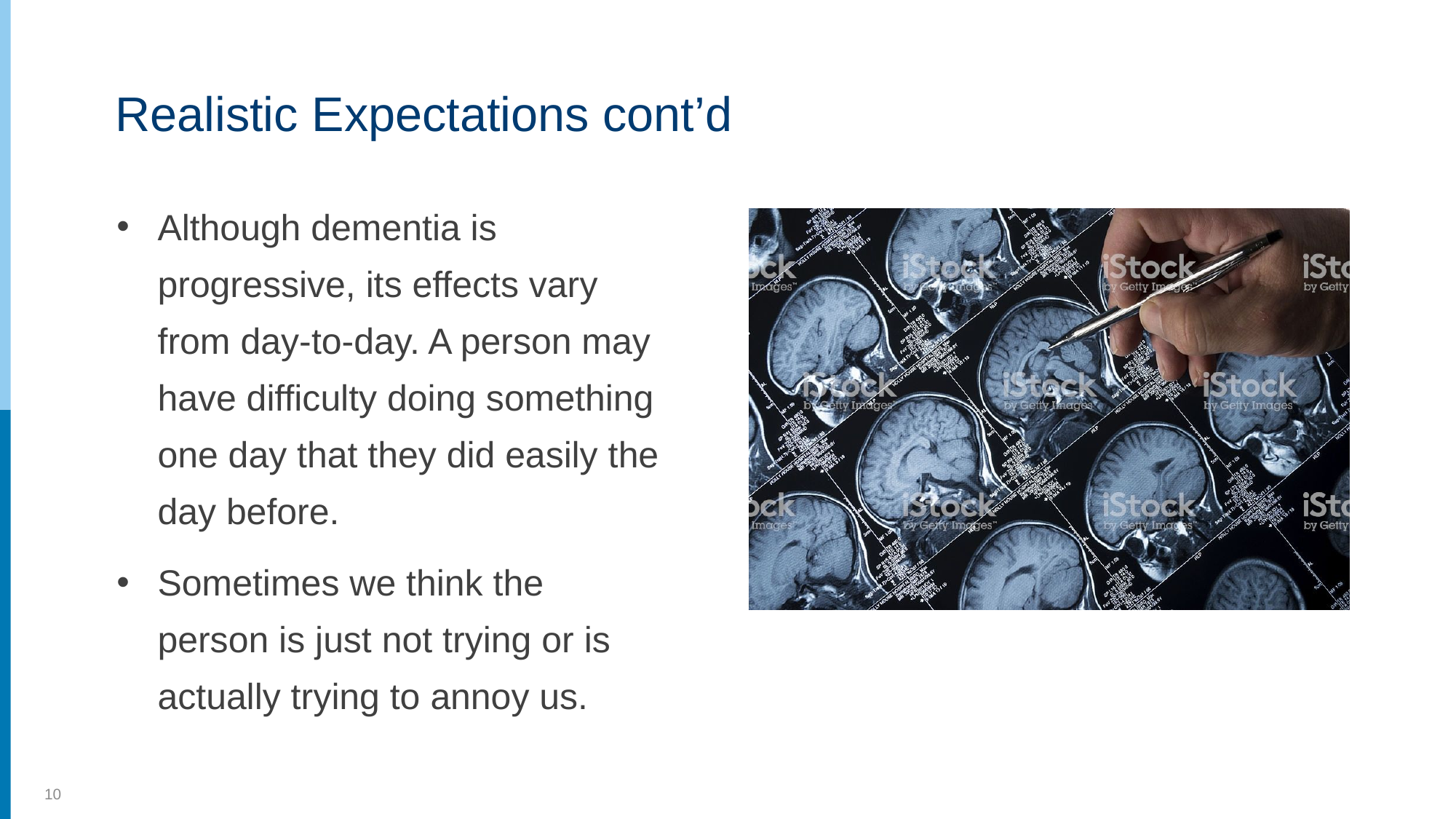

Realistic Expectations cont’d
Although dementia is progressive, its effects vary from day-to-day. A person may have difficulty doing something one day that they did easily the day before.
Sometimes we think the person is just not trying or is actually trying to annoy us.

## Slide 11
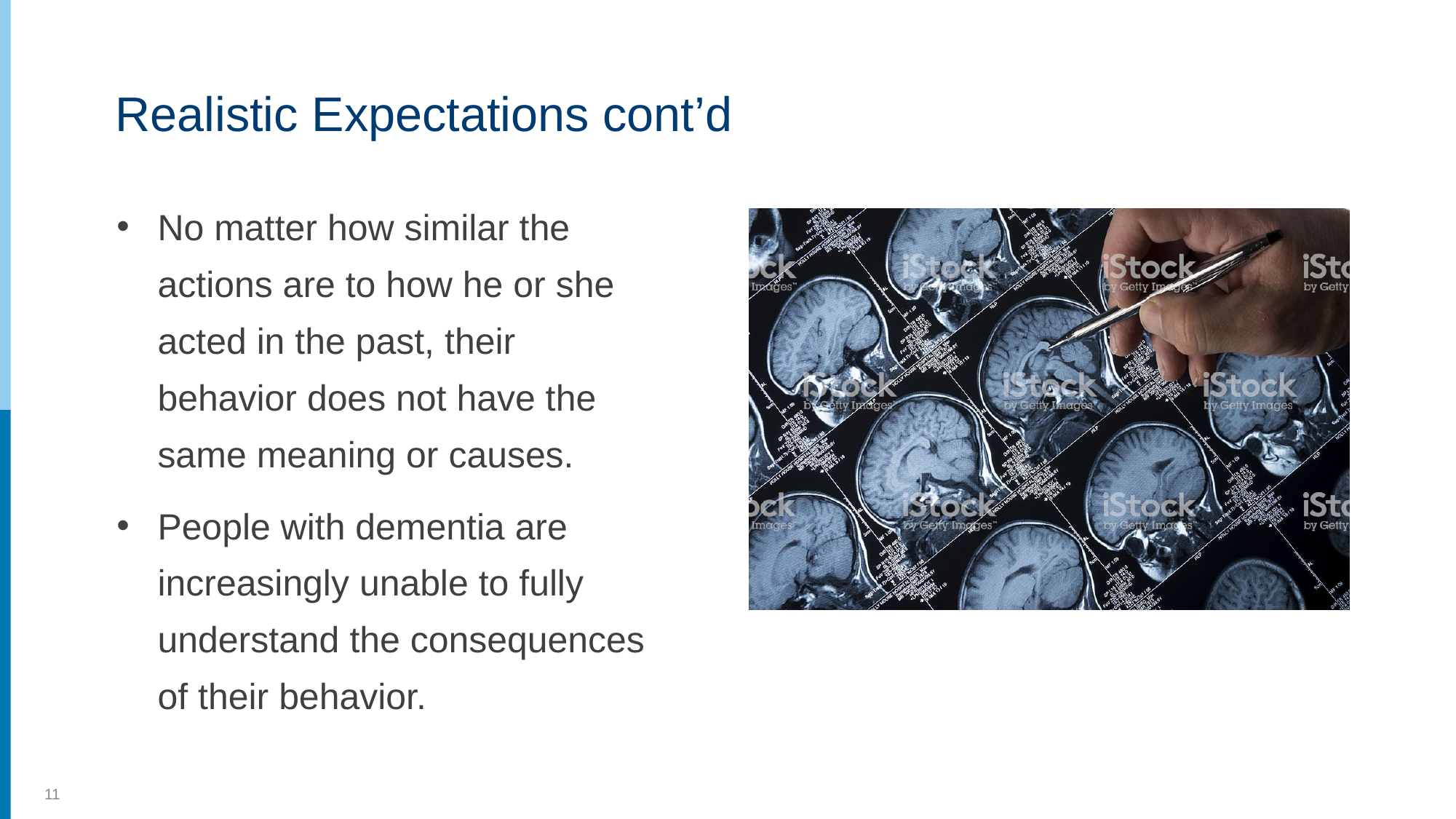

Realistic Expectations cont’d
No matter how similar the actions are to how he or she acted in the past, their behavior does not have the same meaning or causes.
People with dementia are increasingly unable to fully understand the consequences of their behavior.

## Slide 12
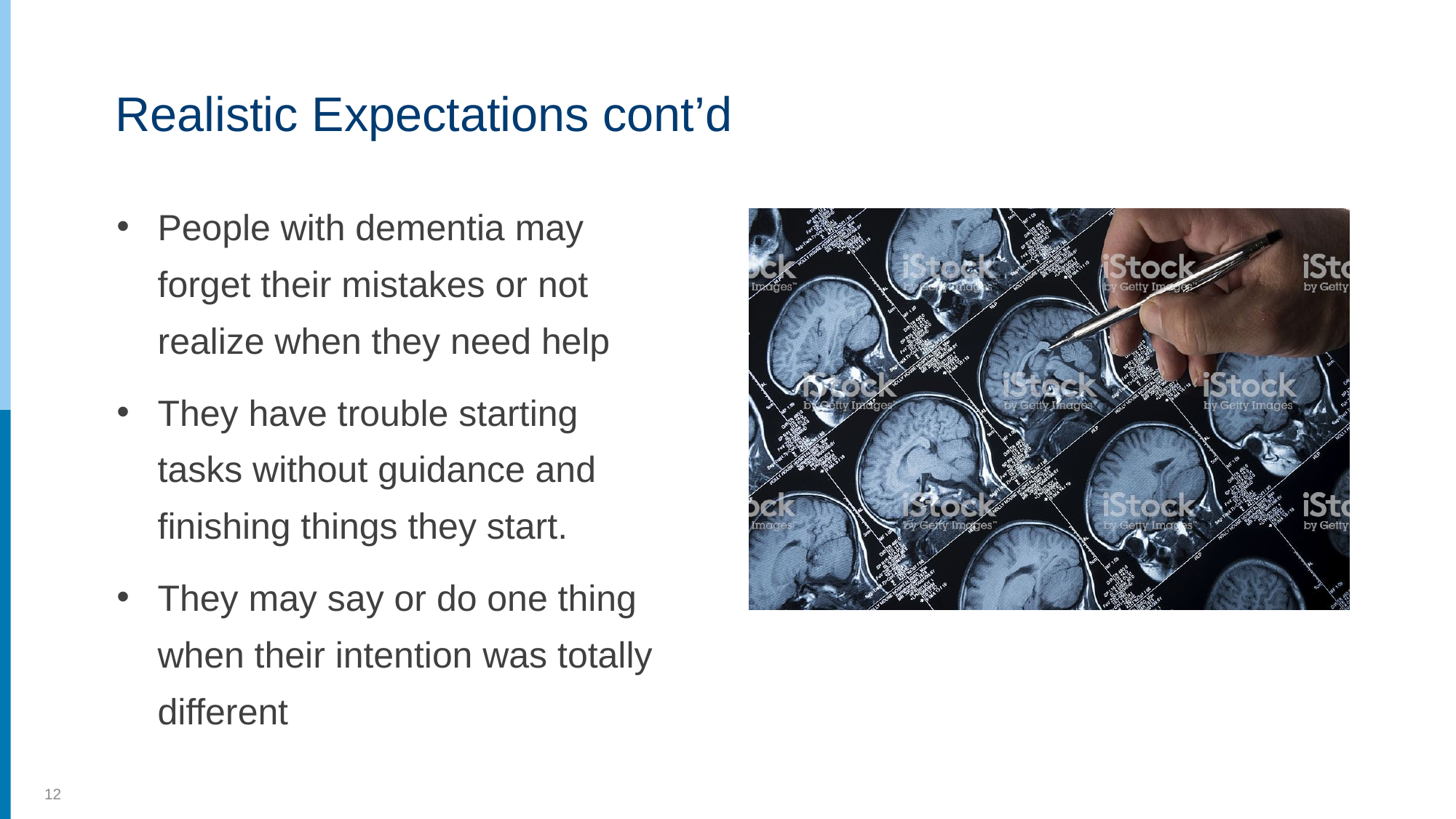

Realistic Expectations cont’d
People with dementia may forget their mistakes or not realize when they need help
They have trouble starting tasks without guidance and finishing things they start.
They may say or do one thing when their intention was totally different

## Slide 13
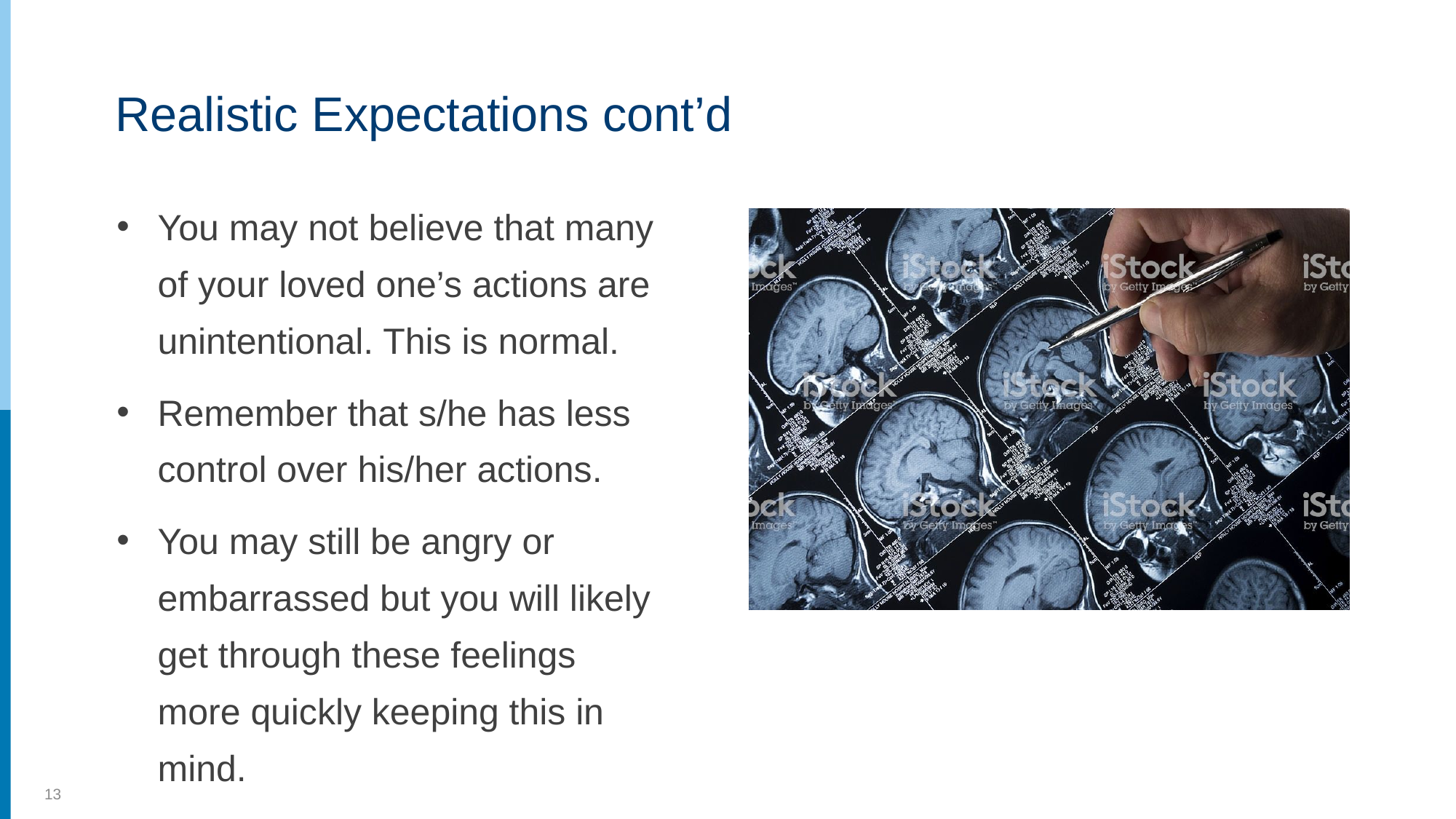

Realistic Expectations cont’d
You may not believe that many of your loved one’s actions are unintentional. This is normal.
Remember that s/he has less control over his/her actions.
You may still be angry or embarrassed but you will likely get through these feelings more quickly keeping this in mind.

## Slide 14
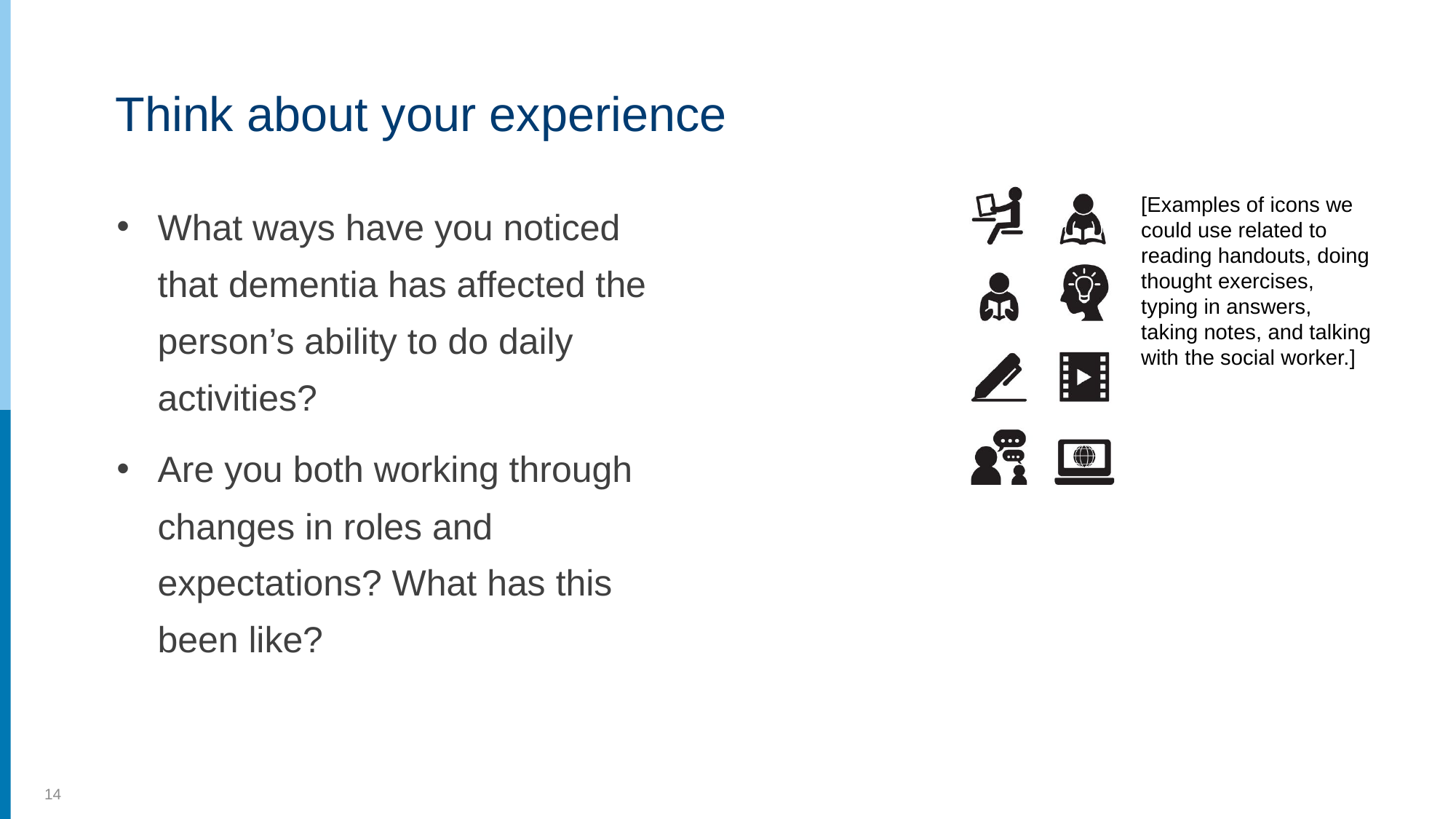

Think about your experience
What ways have you noticed that dementia has affected the person’s ability to do daily activities?
Are you both working through changes in roles and expectations? What has this been like?
[Examples of icons we could use related to reading handouts, doing thought exercises, typing in answers, taking notes, and talking with the social worker.]

## Slide 15
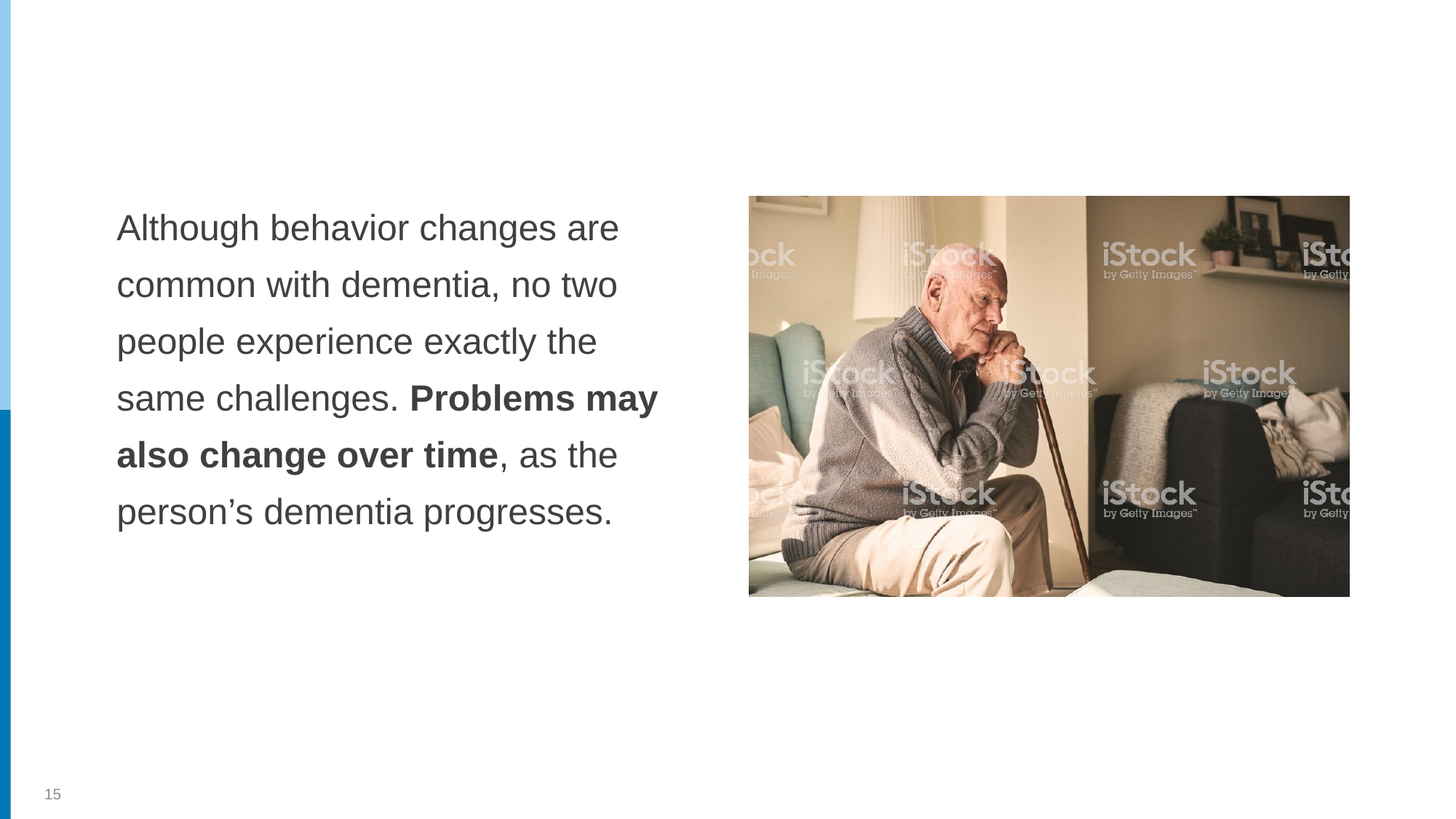

Although behavior changes are common with dementia, no two people experience exactly the same challenges. Problems may also change over time, as the person’s dementia progresses.

## Slide 16
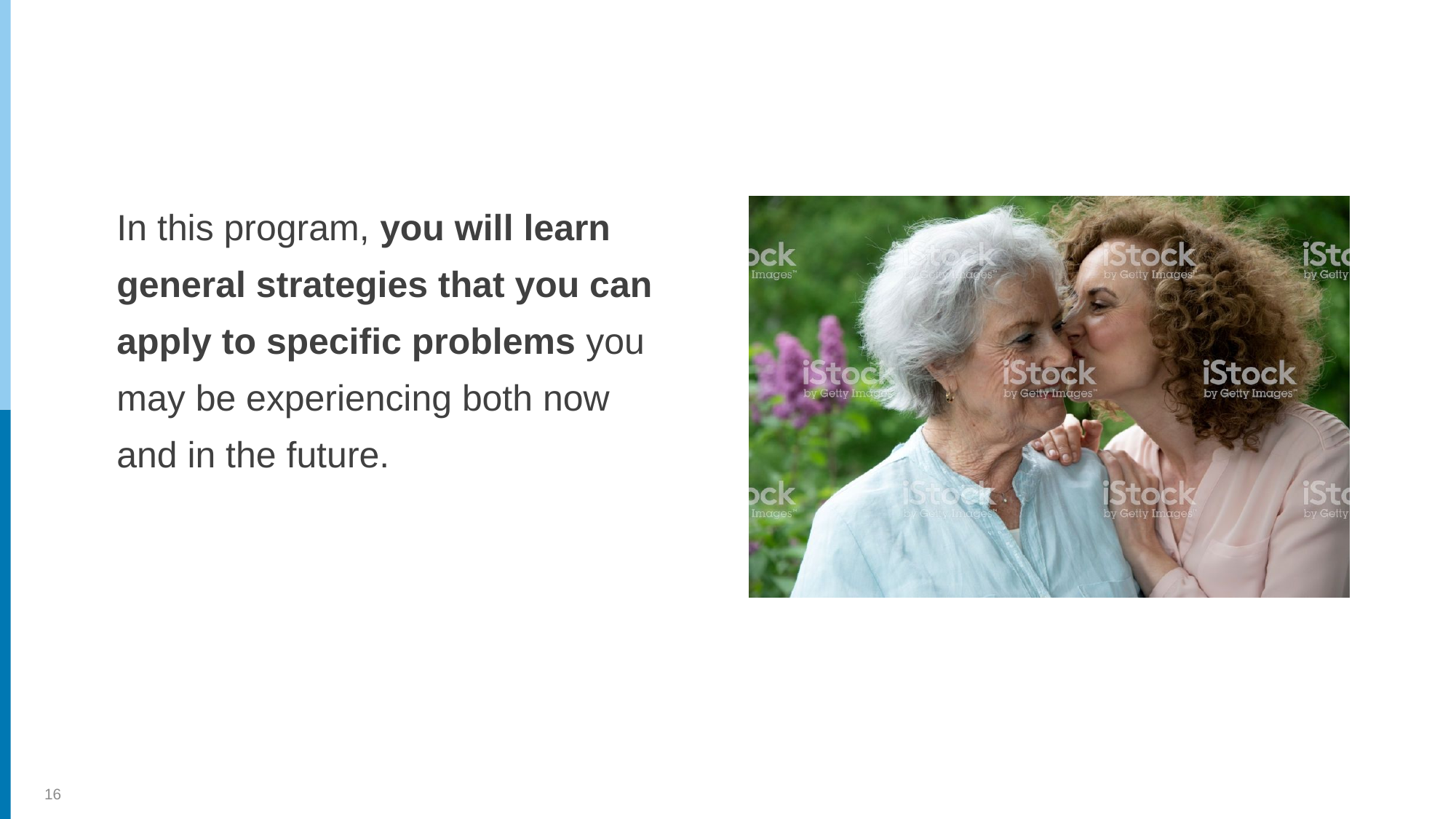

In this program, you will learn general strategies that you can apply to specific problems you may be experiencing both now and in the future.

## Slide 17
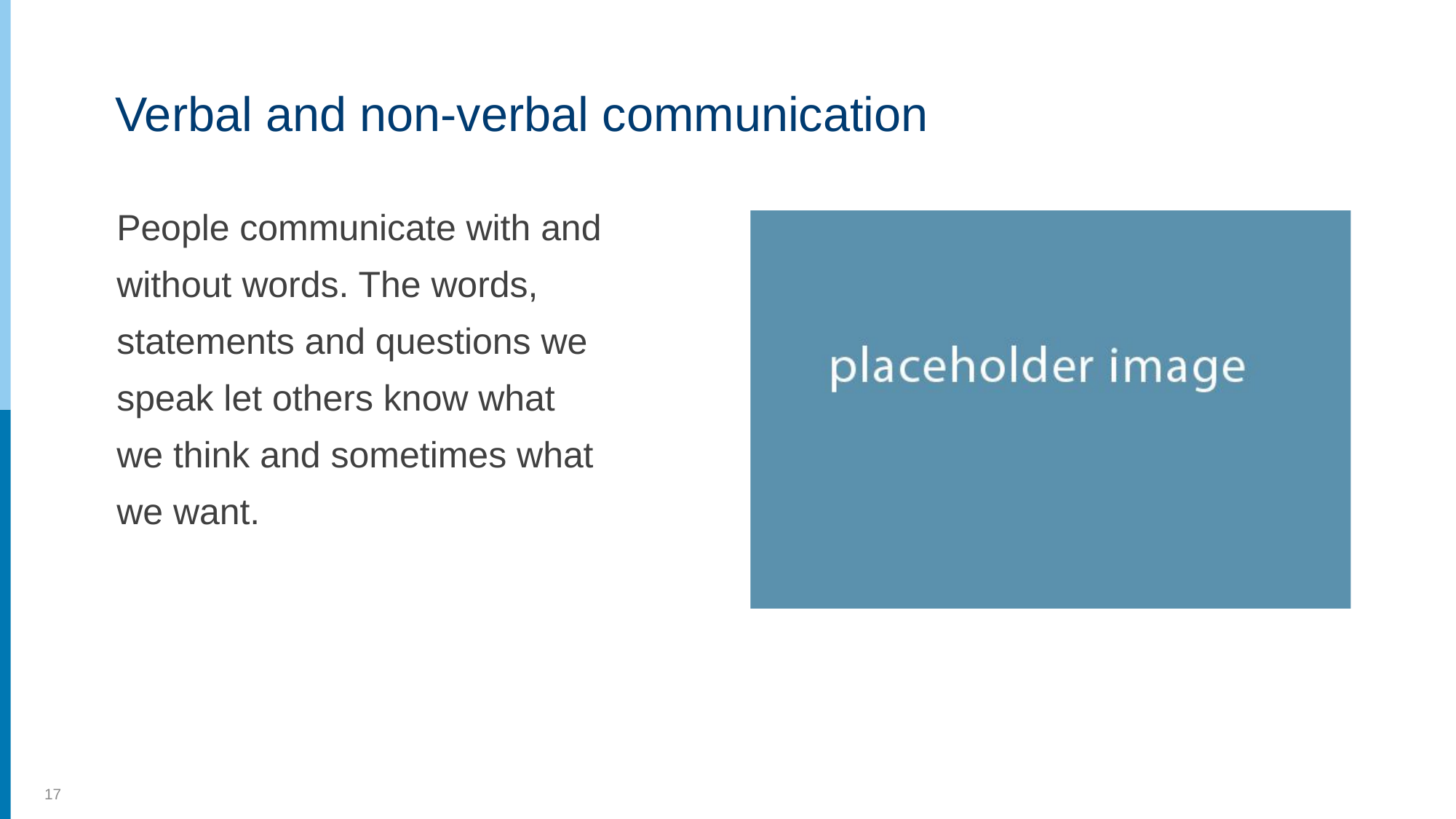

Verbal and non-verbal communication
People communicate with and without words. The words, statements and questions we speak let others know what we think and sometimes what we want.

## Slide 18
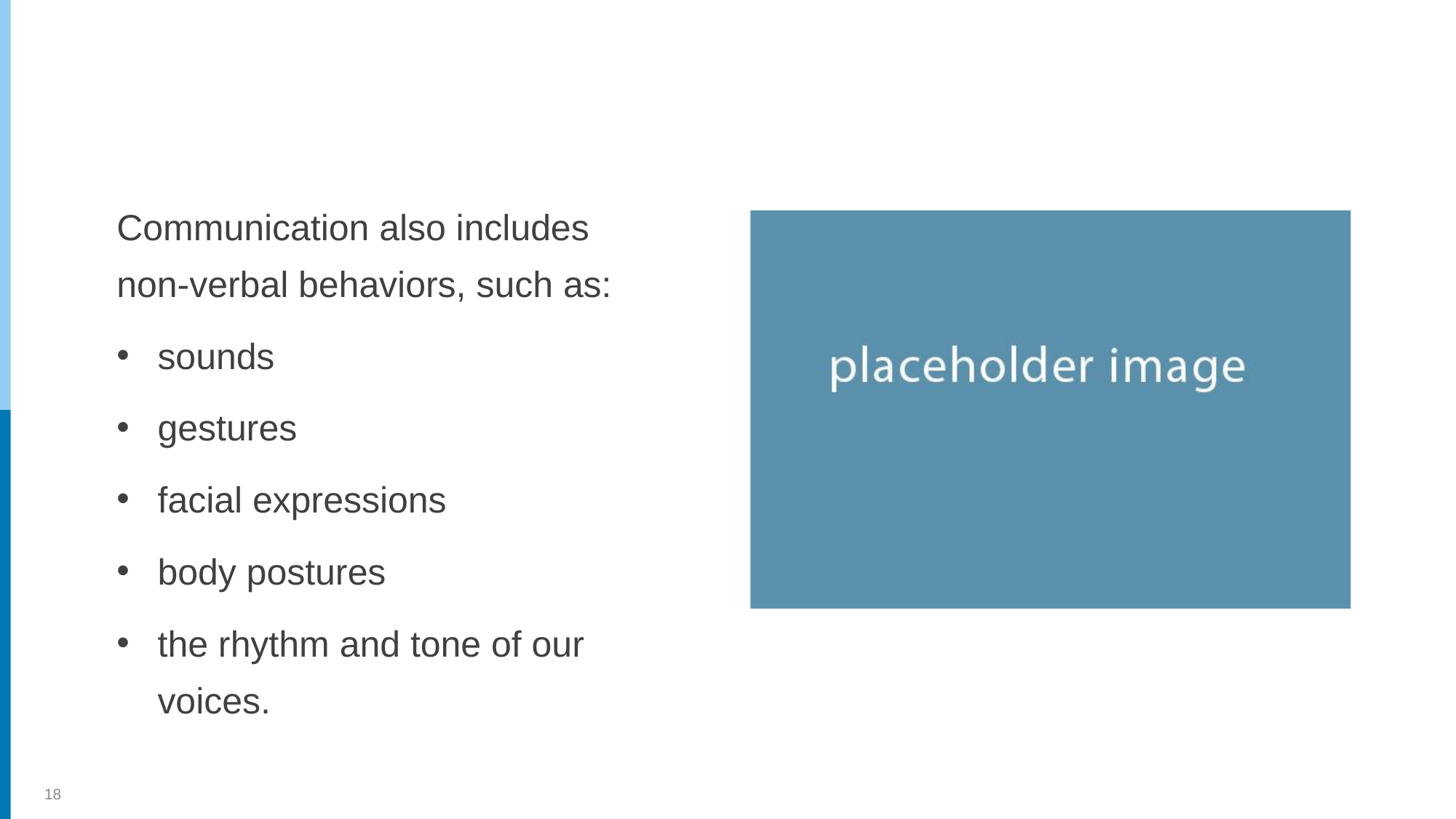

Communication also includes non-verbal behaviors, such as:
sounds
gestures
facial expressions
body postures
the rhythm and tone of our voices.

## Slide 19
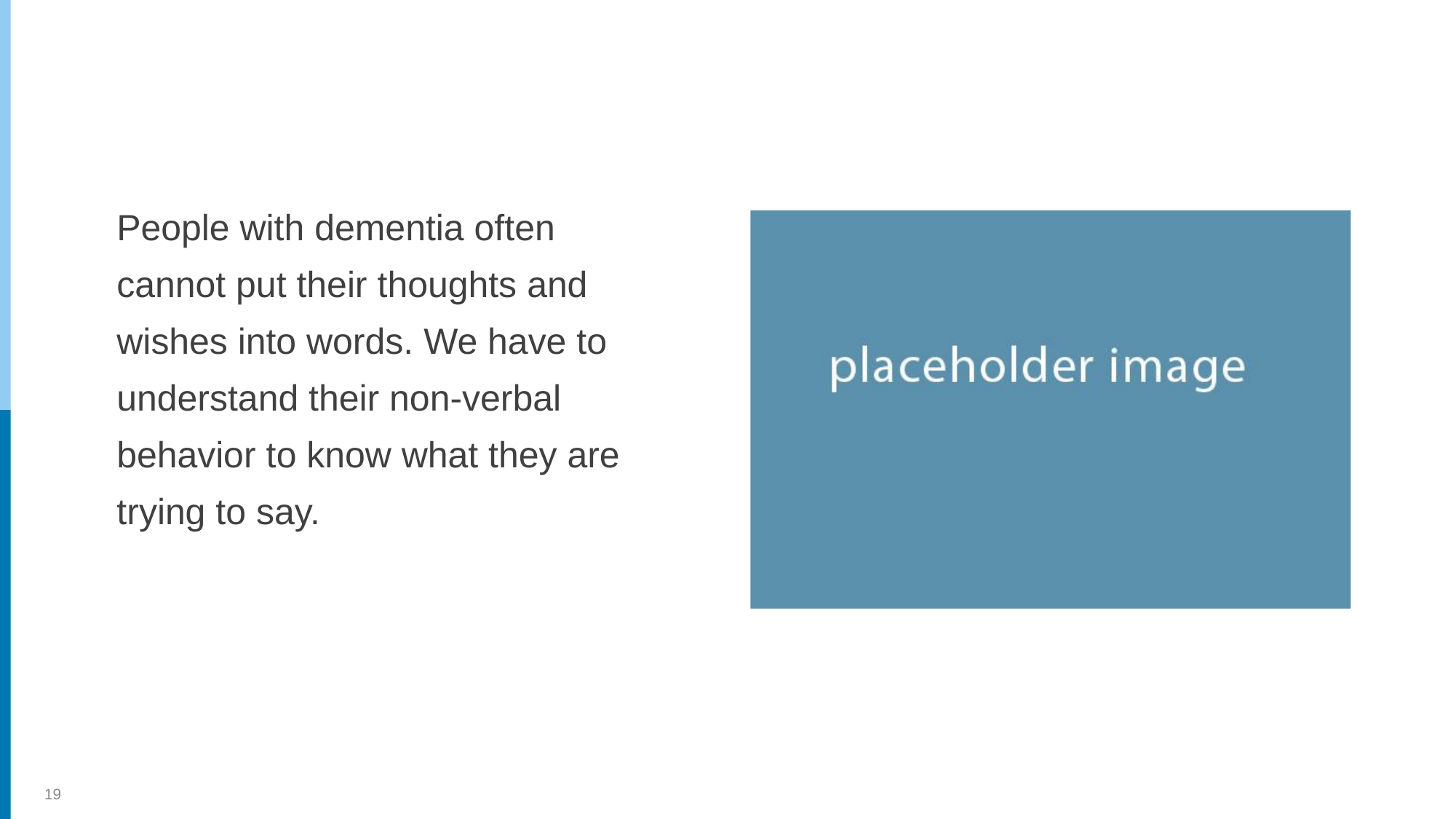

People with dementia often cannot put their thoughts and wishes into words. We have to understand their non-verbal behavior to know what they are trying to say.

## Slide 20
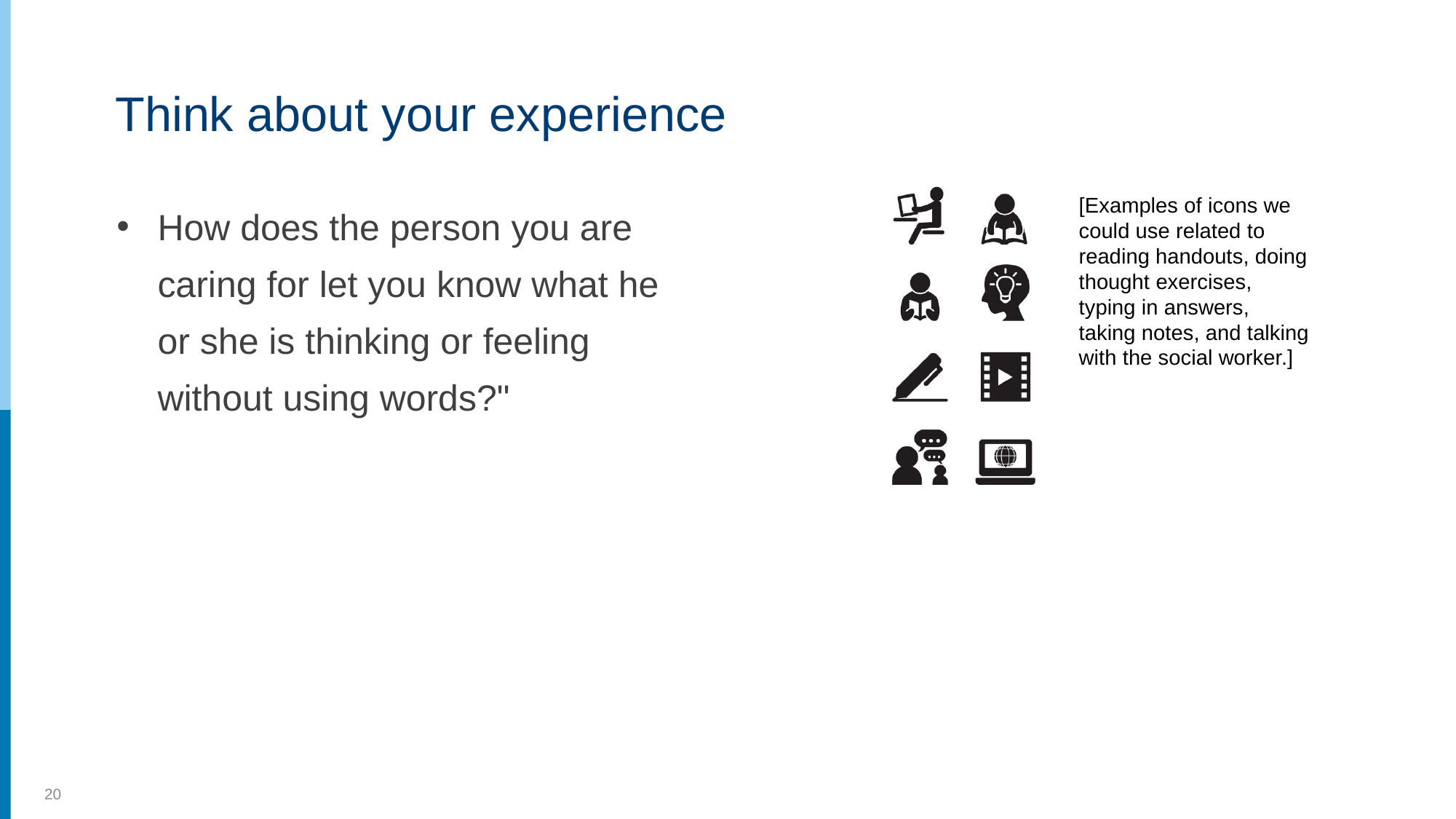

Think about your experience
How does the person you are caring for let you know what he or she is thinking or feeling without using words?"
[Examples of icons we could use related to reading handouts, doing thought exercises, typing in answers, taking notes, and talking with the social worker.]

## Slide 21
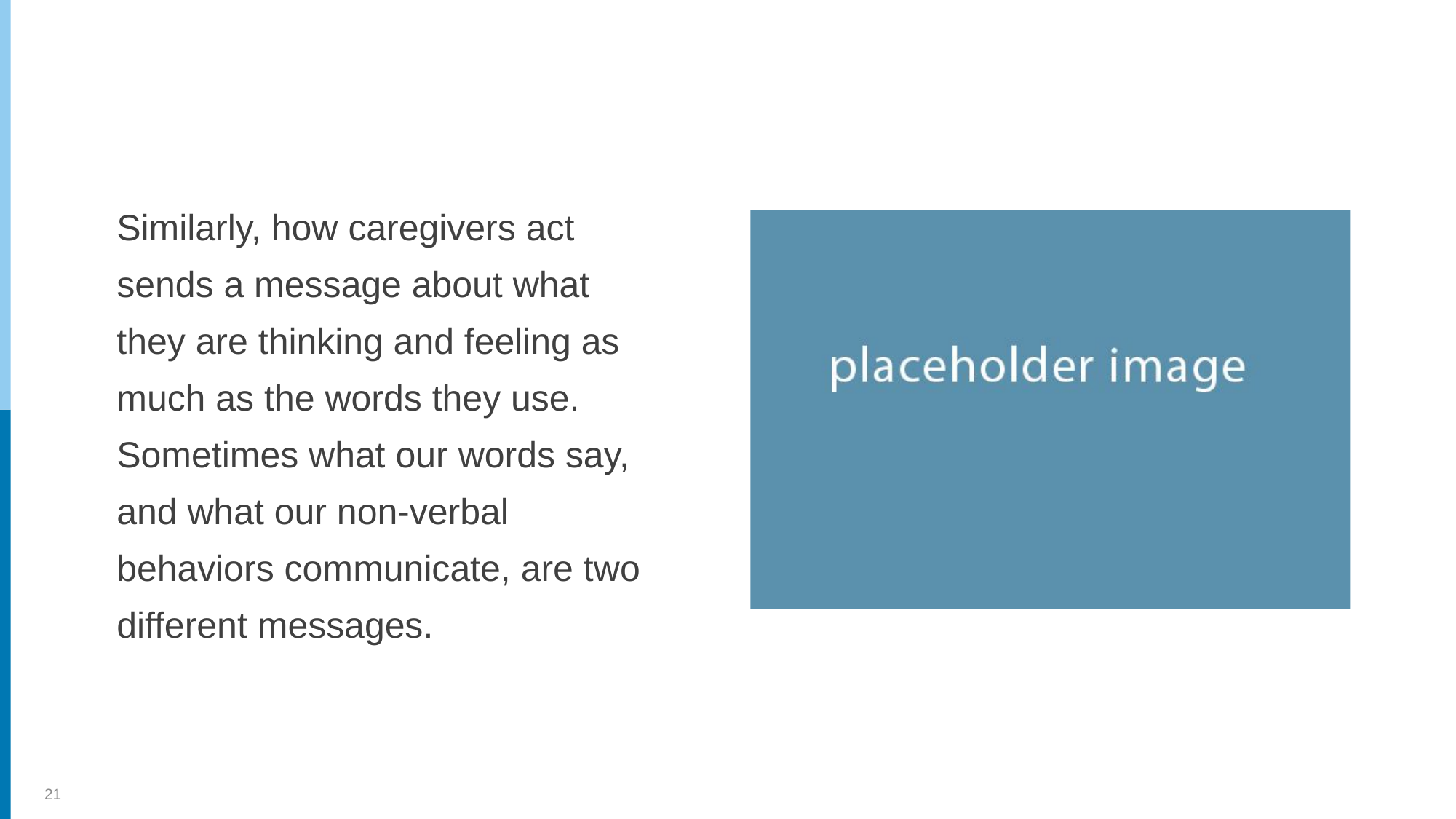

Similarly, how caregivers act sends a message about what they are thinking and feeling as much as the words they use. Sometimes what our words say, and what our non-verbal behaviors communicate, are two different messages.

## Slide 22
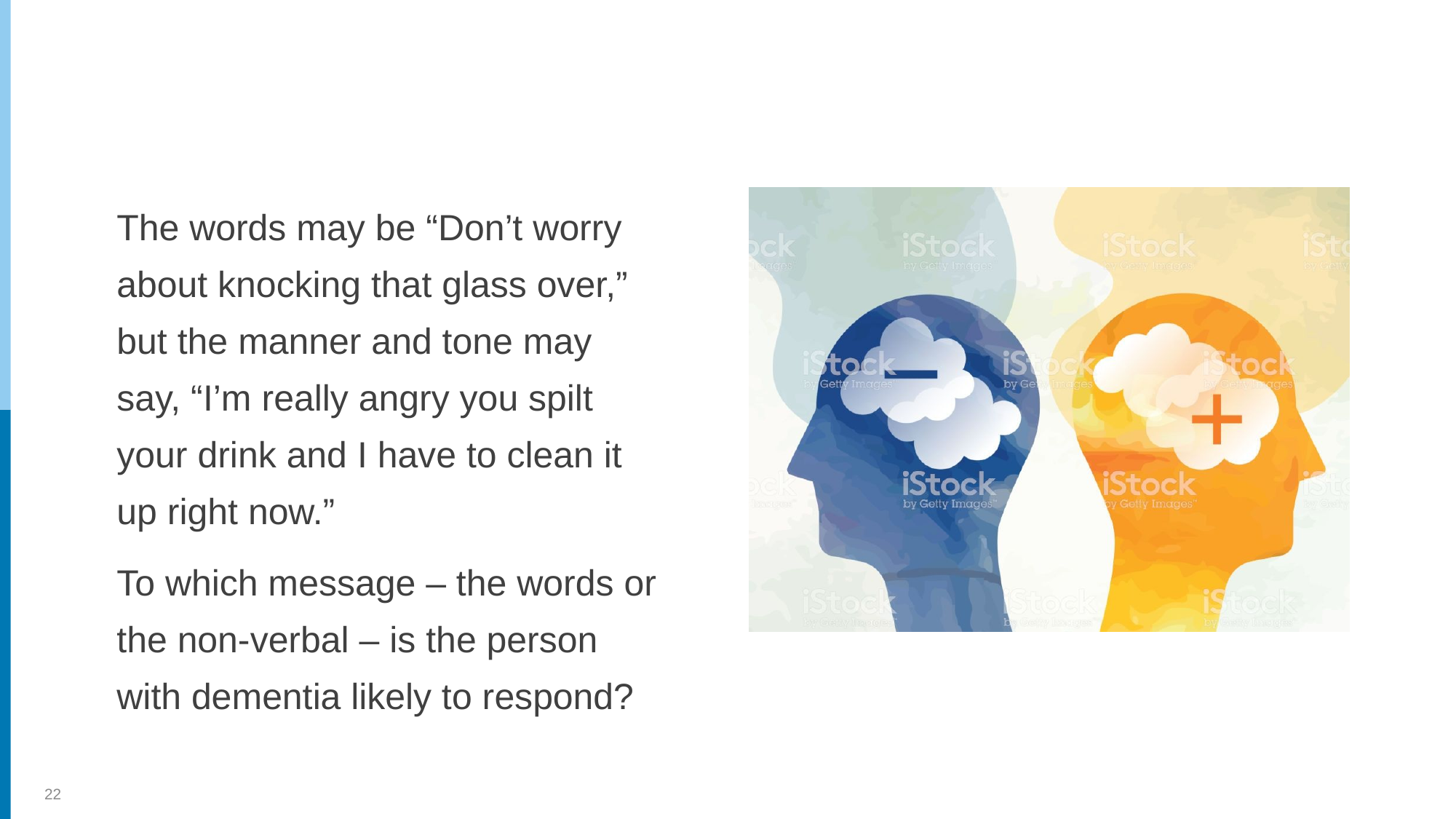

The words may be “Don’t worry about knocking that glass over,” but the manner and tone may say, “I’m really angry you spilt your drink and I have to clean it up right now.”
To which message – the words or the non-verbal – is the person with dementia likely to respond?

## Slide 23
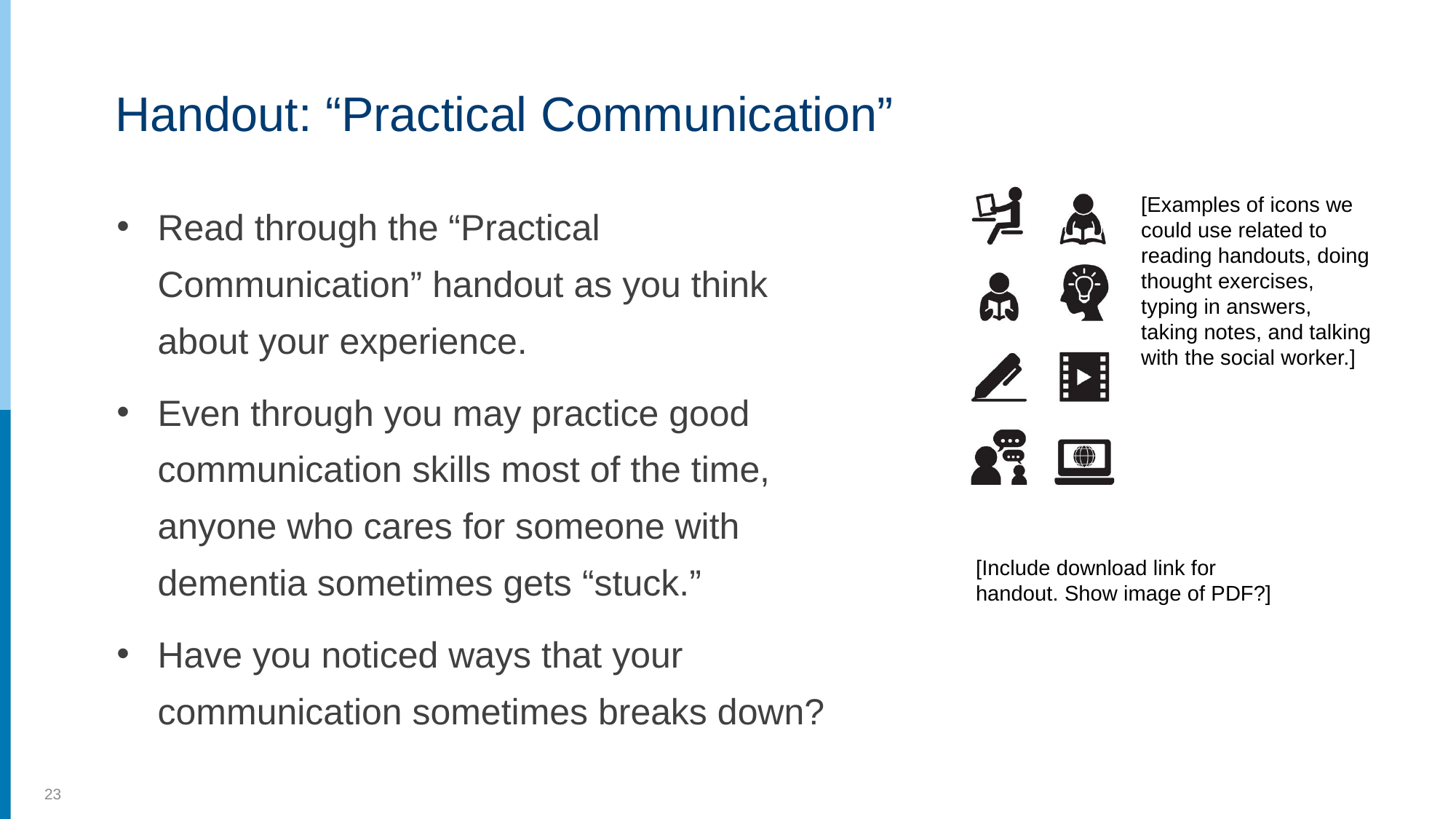

Handout: “Practical Communication”
Read through the “Practical Communication” handout as you think about your experience.
Even through you may practice good communication skills most of the time, anyone who cares for someone with dementia sometimes gets “stuck.”
Have you noticed ways that your communication sometimes breaks down?
[Examples of icons we could use related to reading handouts, doing thought exercises, typing in answers, taking notes, and talking with the social worker.]
[Include download link for handout. Show image of PDF?]

## Slide 24
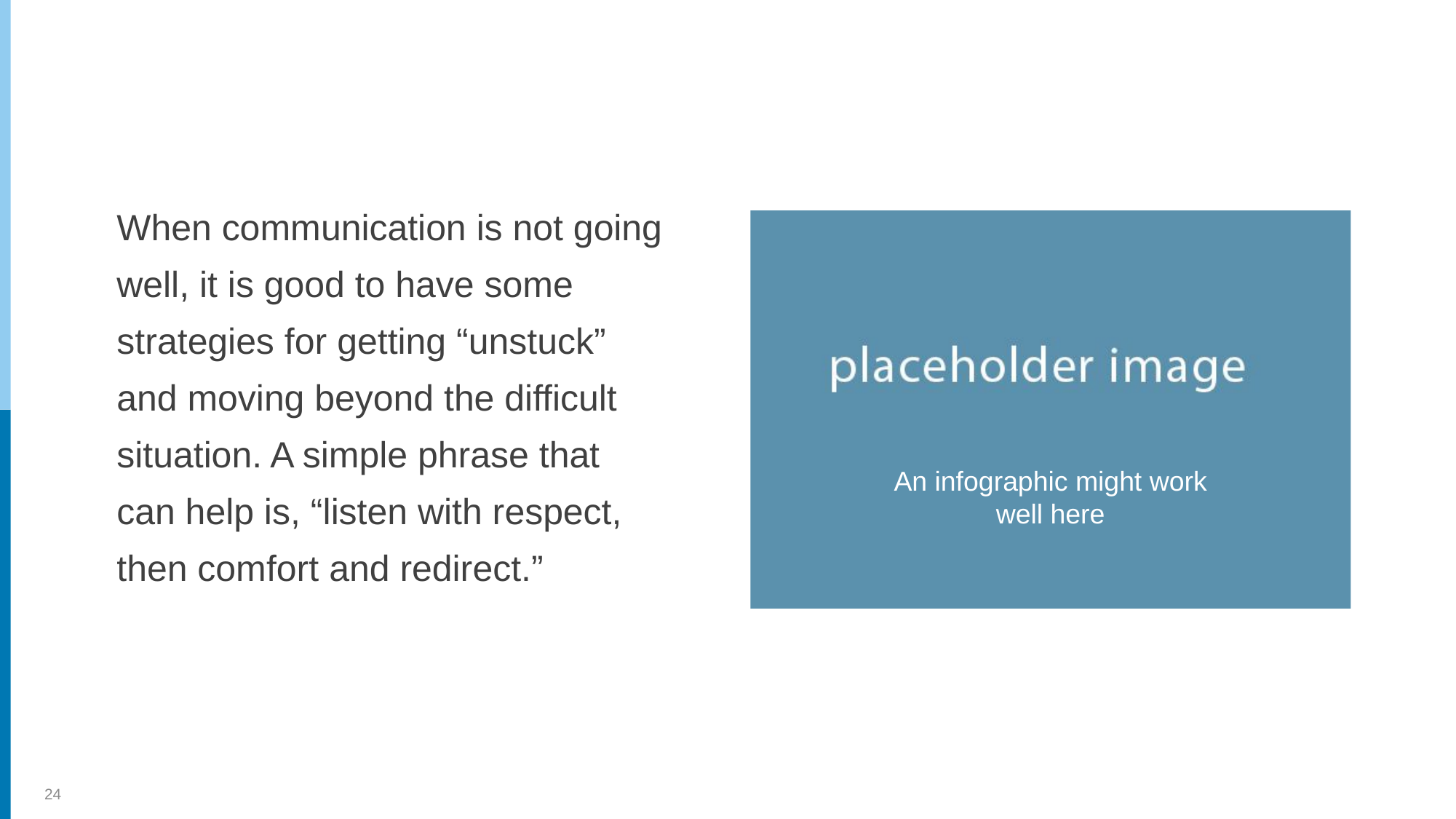

When communication is not going well, it is good to have some strategies for getting “unstuck” and moving beyond the difficult situation. A simple phrase that can help is, “listen with respect, then comfort and redirect.”
An infographic might work well here

## Slide 25
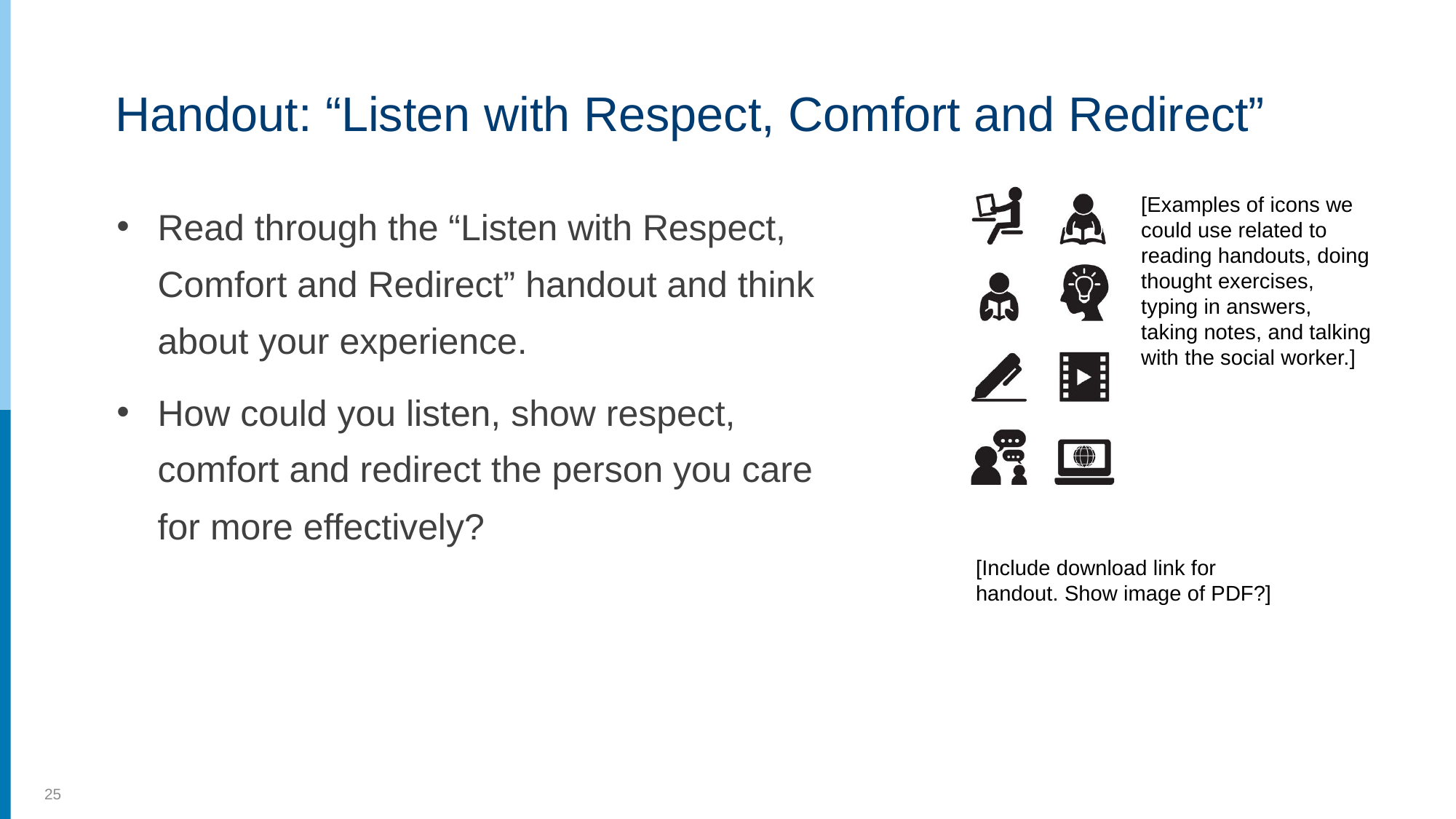

Handout: “Listen with Respect, Comfort and Redirect”
Read through the “Listen with Respect, Comfort and Redirect” handout and think about your experience.
How could you listen, show respect, comfort and redirect the person you care for more effectively?
[Examples of icons we could use related to reading handouts, doing thought exercises, typing in answers, taking notes, and talking with the social worker.]
[Include download link for handout. Show image of PDF?]

## Slide 26
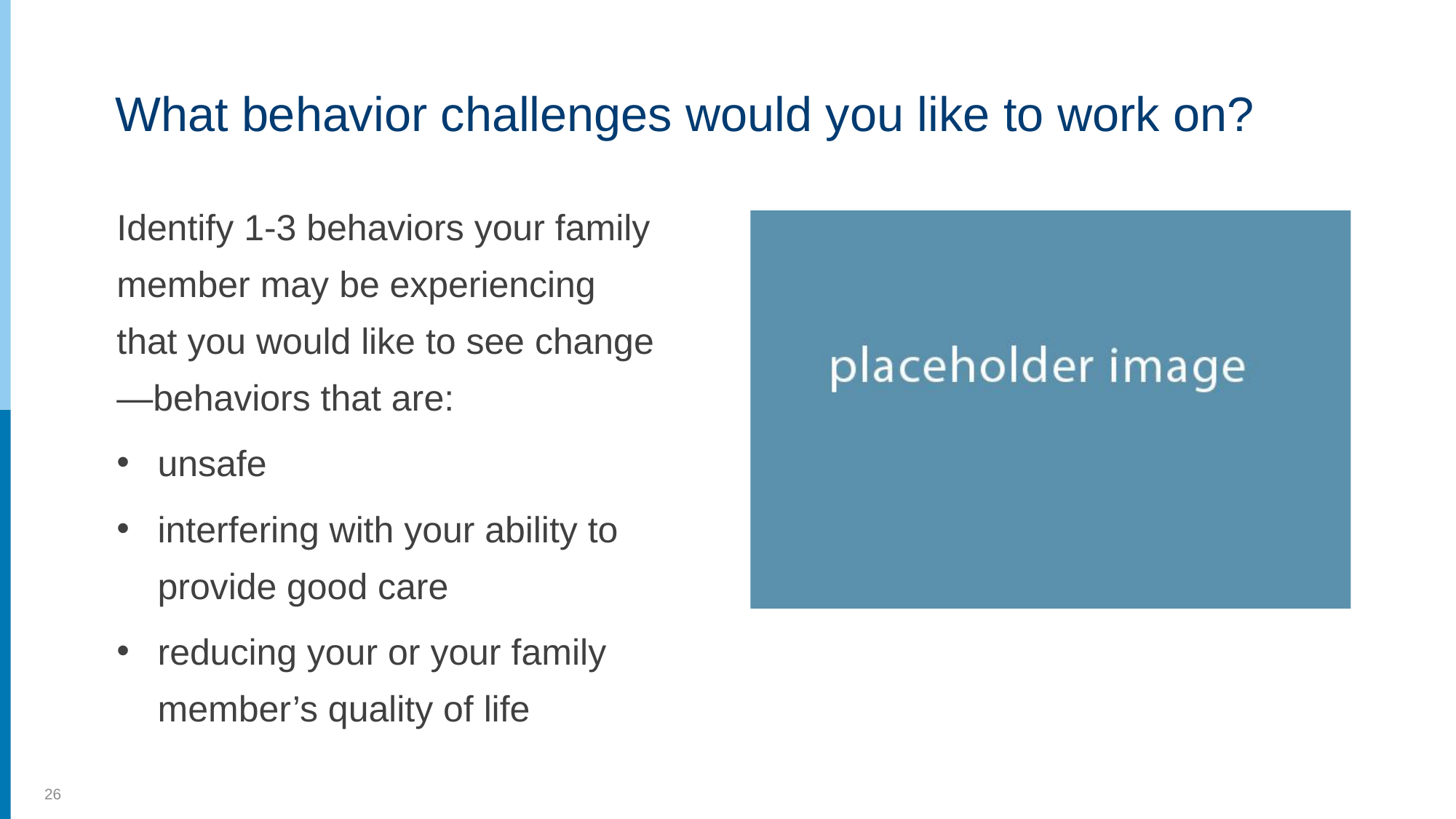

What behavior challenges would you like to work on?
Identify 1-3 behaviors your family member may be experiencing that you would like to see change—behaviors that are:
unsafe
interfering with your ability to provide good care
reducing your or your family member’s quality of life

## Slide 27
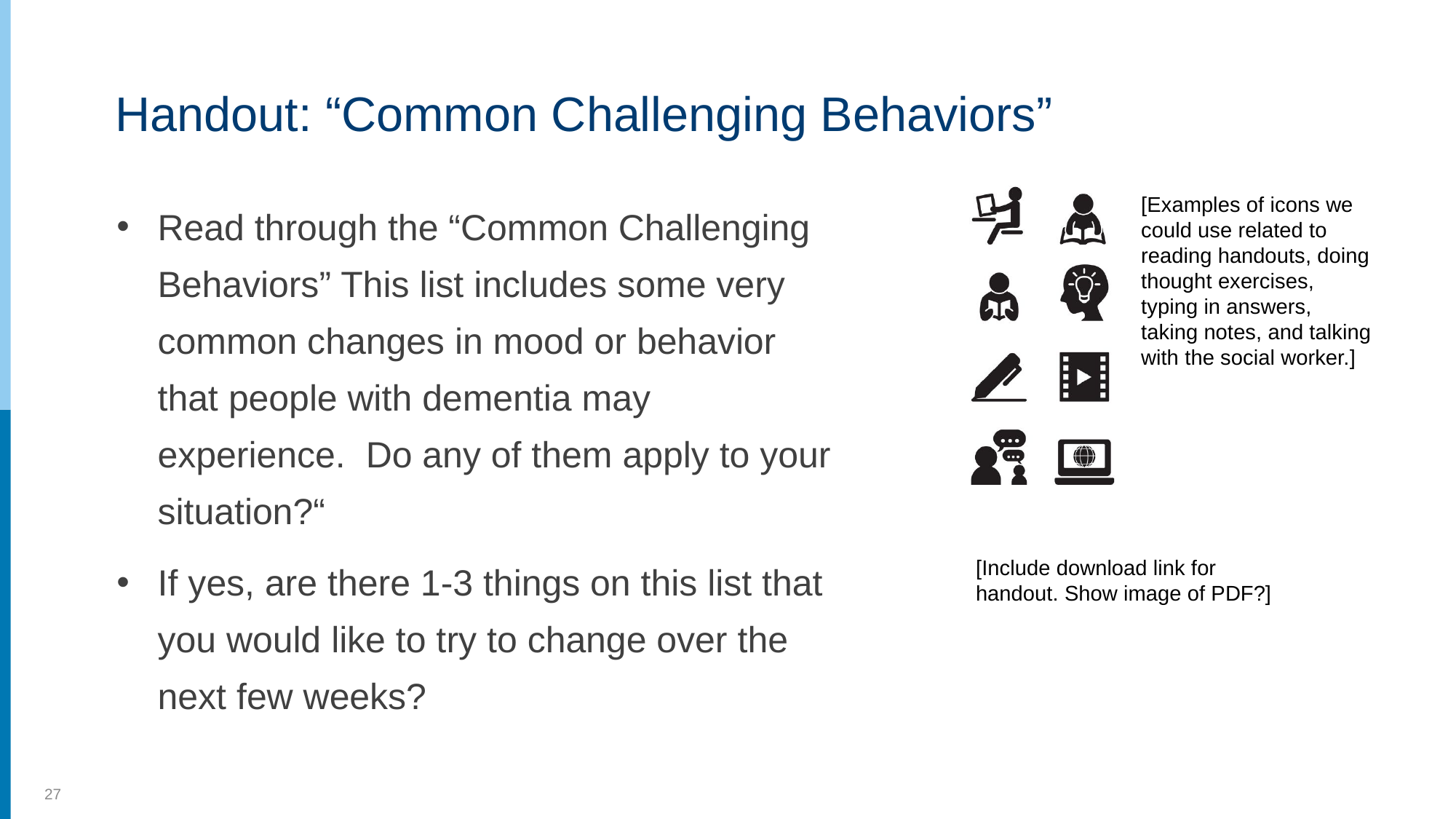

Handout: “Common Challenging Behaviors”
Read through the “Common Challenging Behaviors” This list includes some very common changes in mood or behavior that people with dementia may experience. Do any of them apply to your situation?“
If yes, are there 1-3 things on this list that you would like to try to change over the next few weeks?
[Examples of icons we could use related to reading handouts, doing thought exercises, typing in answers, taking notes, and talking with the social worker.]
[Include download link for handout. Show image of PDF?]

## Slide 28
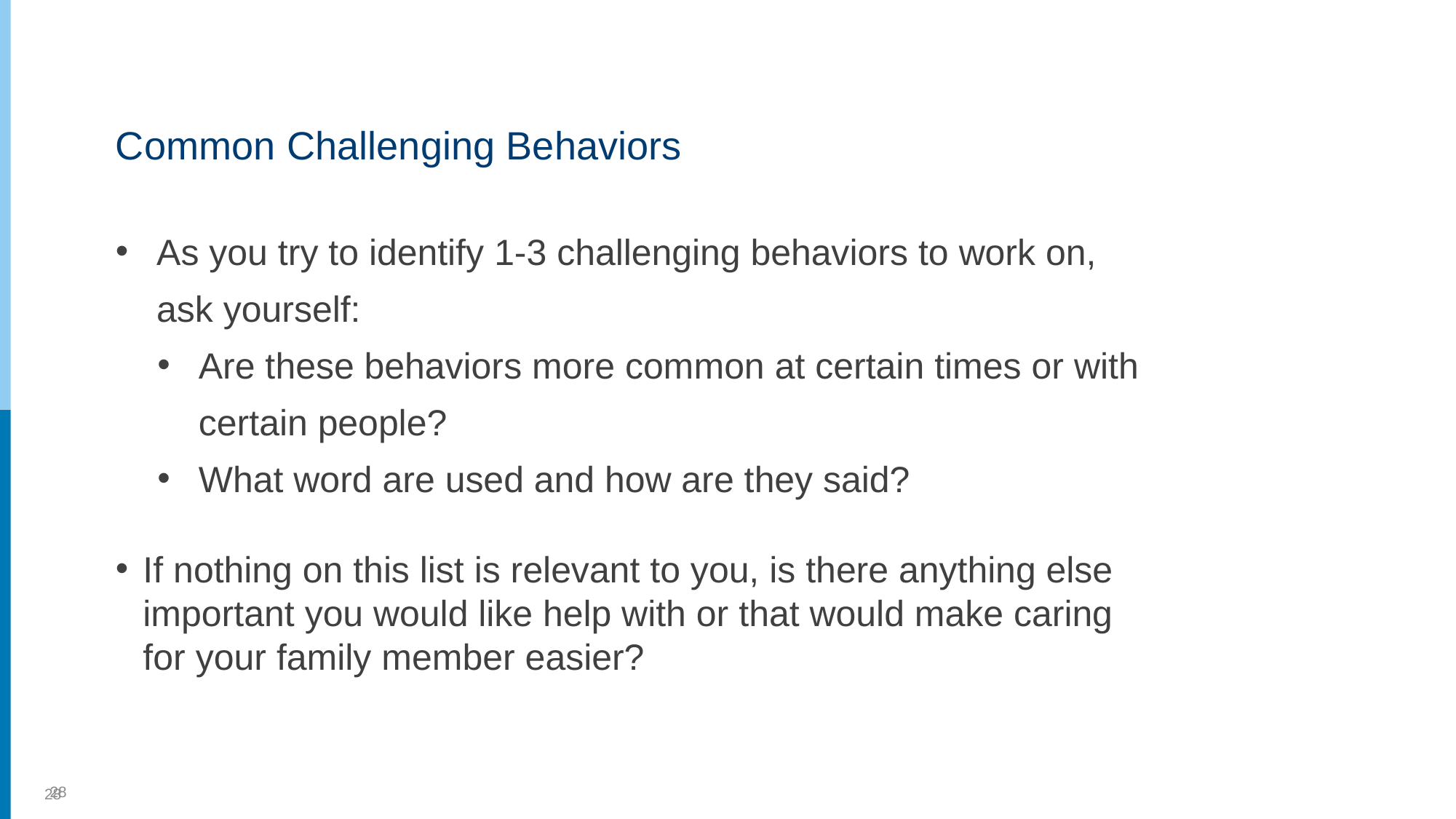

# Common Challenging Behaviors
As you try to identify 1-3 challenging behaviors to work on, ask yourself:
Are these behaviors more common at certain times or with certain people?
What word are used and how are they said?
If nothing on this list is relevant to you, is there anything else important you would like help with or that would make caring for your family member easier?
28

## Slide 29
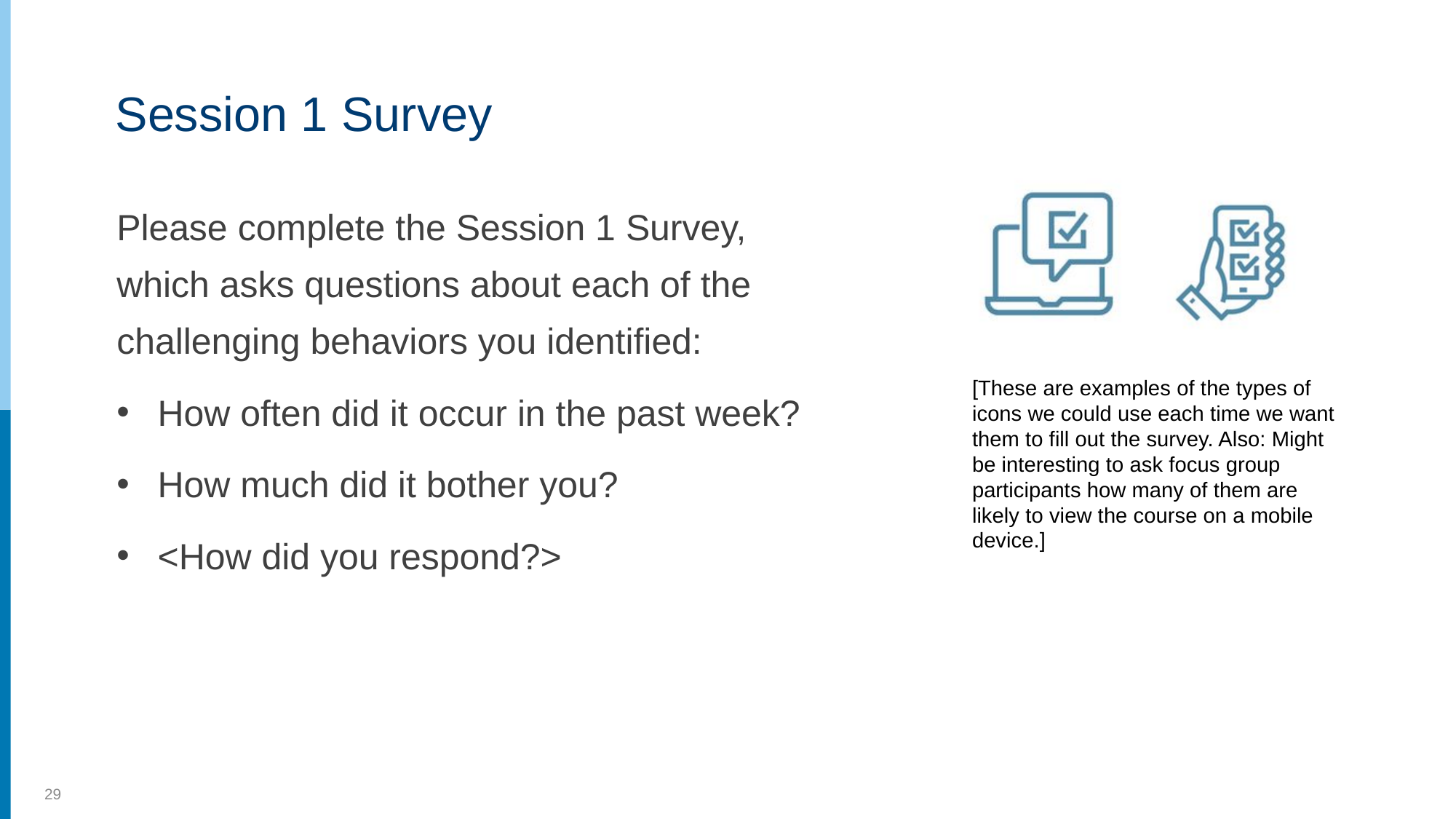

Session 1 Survey
Please complete the Session 1 Survey, which asks questions about each of the challenging behaviors you identified:
How often did it occur in the past week?
How much did it bother you?
<How did you respond?>
[These are examples of the types of icons we could use each time we want them to fill out the survey. Also: Might be interesting to ask focus group participants how many of them are likely to view the course on a mobile device.]

## Slide 30
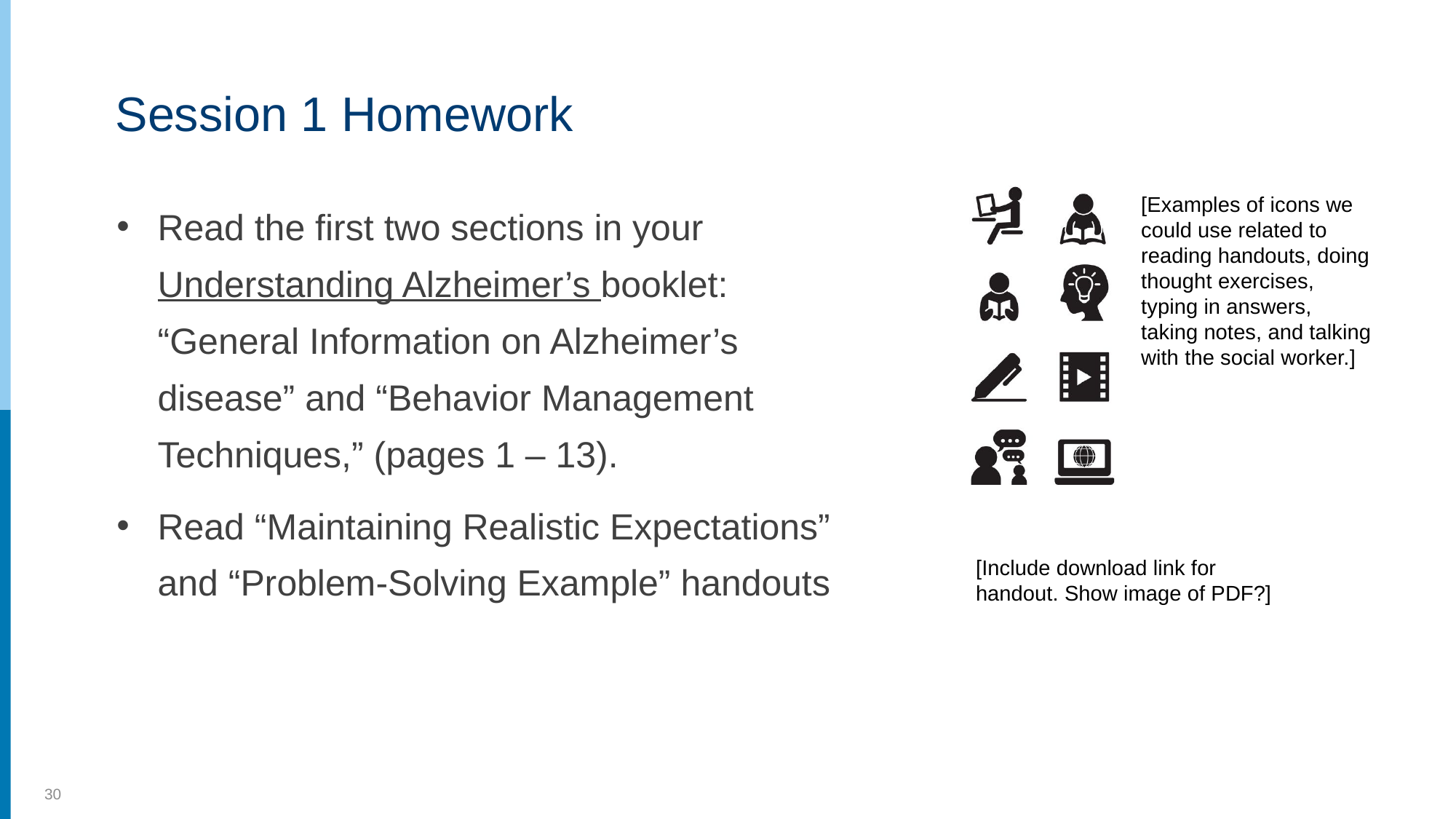

Session 1 Homework
Read the first two sections in your Understanding Alzheimer’s booklet: “General Information on Alzheimer’s disease” and “Behavior Management Techniques,” (pages 1 – 13).
Read “Maintaining Realistic Expectations” and “Problem-Solving Example” handouts
[Examples of icons we could use related to reading handouts, doing thought exercises, typing in answers, taking notes, and talking with the social worker.]
[Include download link for handout. Show image of PDF?]

## Slide 31
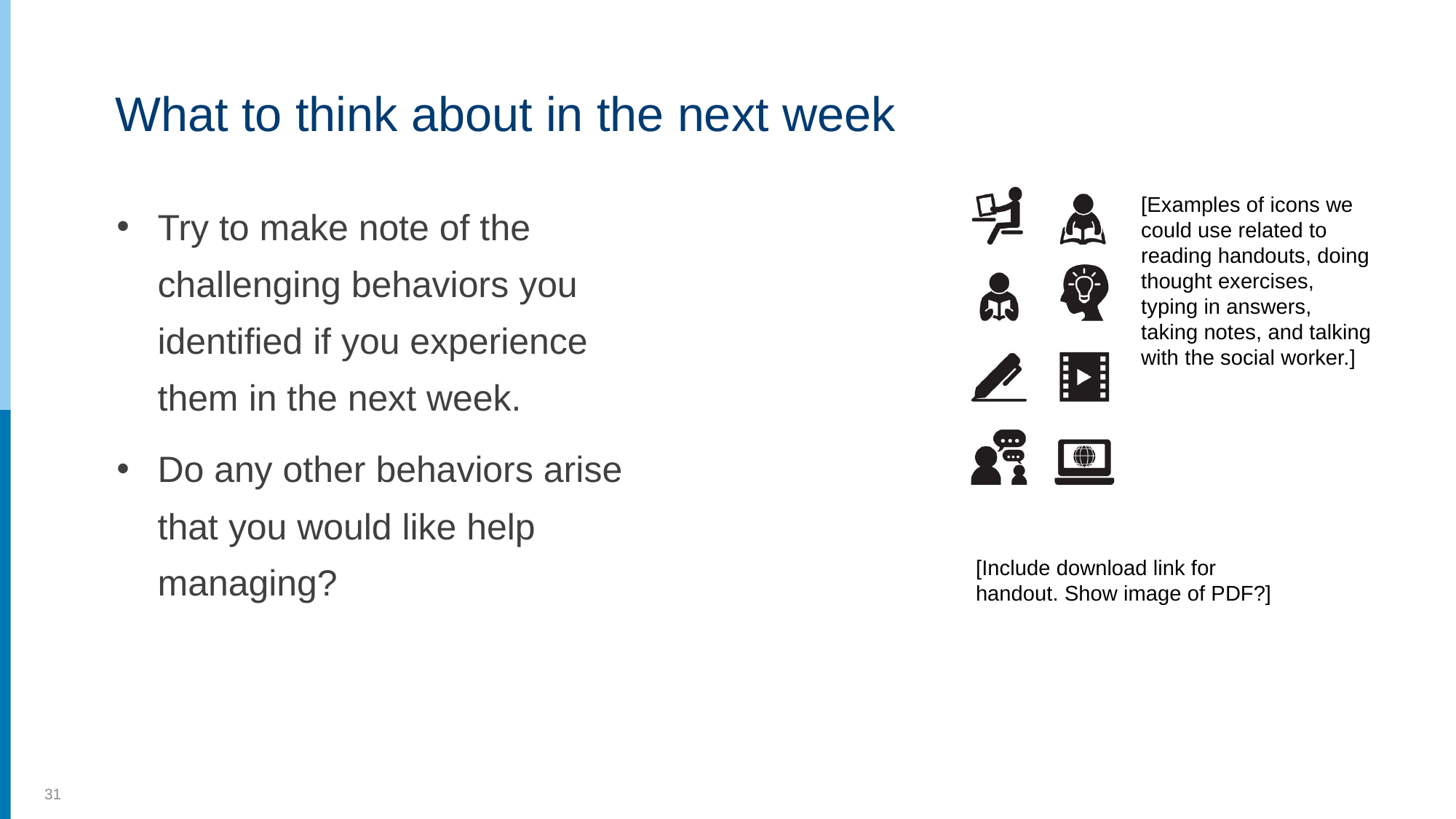

What to think about in the next week
Try to make note of the challenging behaviors you identified if you experience them in the next week.
Do any other behaviors arise that you would like help managing?
[Examples of icons we could use related to reading handouts, doing thought exercises, typing in answers, taking notes, and talking with the social worker.]
[Include download link for handout. Show image of PDF?]
